# Visualization of Inflammation in Experimental Colitis by Magnetic Resonance Imaging Using Very Small Superparamagnetic Iron Oxide Particles

**DOI:** 10.3389/fphys.2022.862212

**Published:** 2022-07-12

**Authors:** Laura Golusda, Anja A. Kühl, Malte Lehmann, Katja Dahlke, Susanne Mueller, Philipp Boehm-Sturm, Jessica Saatz, Heike Traub, Joerg Schnorr, Christian Freise, Matthias Taupitz, Karina Biskup, Véronique Blanchard, Oliver Klein, Ingolf Sack, Britta Siegmund, Daniela Paclik

**Affiliations:** ^1^ Medical Department, Division of Gastroenterology, Infectiology and Rheumatology, Campus Benjamin Franklin, Charité-Universitätsmedizin Berlin, Corporate Member of Freie Universität Berlin and Humboldt-Universität zu Berlin, Berlin, Germany; ^2^ iPATH.Berlin, Campus Benjamin Franklin, Charité-Universitätsmedizin Berlin, Corporate Member of Freie Universität Berlin and Humboldt-Universität zu Berlin, Berlin, Germany; ^3^ Department of Biology, Chemistry and Pharmacy, Institute of Chemistry and Biochemistry, Freie Universität Berlin, Berlin, Germany; ^4^ Department of Experimental Neurology and Center for Stroke Research, Campus Mitte, Charité-Universitätsmedizin Berlin, Corporate Member of Freie Universität Berlin and Humboldt-Universität zu Berlin, Berlin, Germany; ^5^ NeuroCure Cluster of Excellence and Charité Core Facility 7T Experimental MRIs, Campus Mitte, Charité-Universitätsmedizin Berlin, Corporate Member of Freie Universität Berlin and Humboldt-Universität zu Berlin, Berlin, Germany; ^6^ Bundesanstalt für Materialforschung und-prüfung (BAM), Division Inorganic Trace Analysis, Berlin, Germany; ^7^ Department of Radiology-Experimental Radiology, Campus Mitte, Charité-Universitätsmedizin Berlin, Corporate Member of Freie Universität Berlin and Humboldt-Universität zu Berlin, Berlin, Germany; ^8^ Campus Virchow-Klinikum, Institute of Laboratory Medicine, Clinical Chemistry and Pathobiochemistry, Charité-Universitätsmedizin Berlin, Corporate Member of Freie Universität Berlin and Humboldt-Universität zu Berlin, Berlin, Germany; ^9^ BIH-Center for Regenerative Therapies, Campus Virchow-Klinikum, Charité-Universitätsmedizin Berlin, Corporate Member of Freie Universität Berlin and Humboldt-Universität zu Berlin, Berlin, Germany

**Keywords:** magnetic resonance imaging, inflammatory bowel diseases, extracellular matrix, DSS-induced colitis, transfer colitis, very small superparamagnetic iron oxide nanoparticles

## Abstract

Inflammatory bowel diseases (IBD) comprise mainly ulcerative colitis (UC) and Crohn´s disease (CD). Both forms present with a chronic inflammation of the (gastro) intestinal tract, which induces excessive changes in the composition of the associated extracellular matrix (ECM). In UC, the inflammation is limited to the colon, whereas it can occur throughout the entire gastrointestinal tract in CD. Tools for early diagnosis of IBD are still very limited and highly invasive and measures for standardized evaluation of structural changes are scarce. To investigate an efficient non-invasive way of diagnosing intestinal inflammation and early changes of the ECM, very small superparamagnetic iron oxide nanoparticles (VSOPs) in magnetic resonance imaging (MRI) were applied in two mouse models of experimental colitis: the dextran sulfate sodium (DSS)-induced colitis and the transfer model of colitis. For further validation of ECM changes and inflammation, tissue sections were analyzed by immunohistochemistry. For in depth *ex-vivo* investigation of VSOPs localization within the tissue, Europium-doped VSOPs served to visualize the contrast agent by imaging mass cytometry (IMC). VSOPs accumulation in the inflamed colon wall of DSS-induced colitis mice was visualized in T_2_* weighted MRI scans. Components of the ECM, especially the hyaluronic acid content, were found to influence VSOPs binding. Using IMC, co-localization of VSOPs with macrophages and endothelial cells in colon tissue was shown. In contrast to the DSS model, colonic inflammation could not be visualized with VSOP-enhanced MRI in transfer colitis. VSOPs present a potential contrast agent for contrast-enhanced MRI to detect intestinal inflammation in mice at an early stage and in a less invasive manner depending on hyaluronic acid content.

## Introduction

The two main forms of inflammatory bowel diseases (IBD) are ulcerative colitis (UC) and Crohn´s disease (CD). Both are live-long threats with recurring disease activity. IBD patients present with a broad spectrum of clinical symptoms including (bloody) diarrhea and weight loss, often accompanied by fever and abdominal cramps (Dignass et al., 2011; Preiss et al., 2014). In CD, the entire gastrointestinal tract can be affected, here 30–40% of the patients suffer of small intestinal inflammation (Strobel et al., 2011), with the terminal ileum being mainly affected. In up to 30% of CD patients, the inflammation is restricted to the colon (Mills and Stamos 2007) characterized by a chronic transmural inflammation with a segmental pattern. In UC, the inflammation is limited to the colonic mucosa and submucosa and spreads continuously from the rectum to the proximal colon (Dignass et al., 2011; Preiss et al., 2014). The incidence and prevalence of IBD are still increasing worldwide with approximately 0.2% of the European population affected (Zhao et al., 2021). Overall, 5–25% of IBD cases develop during childhood or adolescence, whereas 10–15% of patients with IBD will receive their diagnosis >60 years of age (Ruel et al., 2014).

One common complication during CD progression is intestinal fibrosis and subsequent development of strictures caused by an altered extracellular matrix (ECM) composition. Activation of ECM-producing cells together with an imbalance of ECM-constructing and -degrading components, such as matrix metalloproteinase (MMP) and tissue inhibitors of metalloproteinase (TIMP) lead to massive intestinal fibrosis (Giuffrida et al., 2016). Over 50% of CD patients and up to 11% of UC patients experience fibrostenotic complications (Rieder et al., 2017), and it is still unclear whether the ECM modulation is a direct consequence of chronic inflammation or if it is an early event boosting the inflammatory process. So far, there are no reliable biomarkers available to predict early events of intestinal fibrosis (Steiner et al., 2021). Non-invasive techniques like the detection of microbial products, growth factors, gene polymorphisms, micro RNA (miRNA) or ECM products are not specific enough to clearly assess the risk to develop fibrosis (Giuffrida et al., 2016). Within the clinic, magnetic resonance imaging (MRI) is the technique of choice to image fibrotic strictures in IBD. Due to the life-long disease course patients experience multiple MRI scans, each time accompanied by the exposure to gadolinium-based contrast agents. However, recent reports of long-term gadolinium depositions in brain, bones and skin of patients questions the safety of gadolinium-based contrast agents and ask for a more secure alternative (White et al., 2006; Kanda et al., 2015; Robles Cartagena et al., 2015; Flood et al., 2017; Wang et al., 2019; Jacobi et al., 2021). A possible alternative for imaging inflammation is an iron oxide-based contrast agent, called very small superparamagnetic iron oxide particles (VSOPs). VSOPs are currently studied to visualize inflammatory sites in diseases including multiple sclerosis and atherosclerosis as accumulation induced a decreased signal in T_2_/T_2_^*^ weighted MRI post i. v. injection (Wagner et al., 2013; Poller et al., 2018; Millward et al., 2019; Besenhard et al., 2021; Silva et al., 2021). Recent studies suggest that VSOP specifically bind to glycosaminoglycans (GAGs), which are present to a high amount in the ECM (Berndt et al., 2017). Heparin/heparan sulfate, keratan sulfate and chondroitin sulfate/dermatan sulfate are sulfated-GAGs (s-GAGs) while hyaluronic acid (HA) is the only non-sulfated GAG (Frantz et al., 2010; Derkacz et al., 2021a). Changes in the ECM during intestinal inflammation lead to altered synthesis of GAGs and can result in an altered binding capacity of VSOP to the ECM and VSOP-uptake by immune cells in inflamed tissues (Millward et al., 2013; Berndt et al., 2017). Especially dysregulated production of HA is associated with several inflammatory diseases, including IBD (Petrey and de la Motte, 2019). During intestinal inflammation HA is cleaved to lower molecular weight HA chains, contributing to recruitment of mononuclear cells and inflammatory processes (Derkacz et al., 2021b). Furthermore, the transformed vascular ECM enhances the adhesion and extravasation of immune cells like macrophages at the sites of inflammation (Petrey and de la Motte, 2017). This study aimed to investigate the visualization of intestinal inflammation and the early detection of changes in ECM components by using VSOPs in MRI.

## Materials and Methods

### Animals

Inbred wildtype (WT) C57/Bl6J mice were obtained from Charles River (Sulzfeld, Germany) and housed under conventional conditions at the animal facilities of the Research Institute for Experimental Medicine (FEM, Charité - Universitätsmedizin Berlin). Recombination activating gene 1-deficient mice (*Rag* ^*1tm1Mom*^) on a C57BL/6 background were purchased from Jackson Laboratory (Bar Harbor, United States) and bred under SPF conditions at the FEM. All animals were kept in polycarbonate cages and had free access to sterile standard chow and drinking water. All experiments were performed in accordance with the German legislation on the protection of animals and approved by the local authorities (Landesamt für Gesundheit und Soziales, registration number G0422/17).

### Colitis Induction

The study design is summarized in Supplementary Figure S1. Colitis was induced in C57/Bl6J female WT mice by providing 3% dextran sulfate sodium (DSS; MW 36,000–50,000, MP Biomedicals, Santa Ana, CA, United States) via the drinking water from day 0–six followed by pure drinking water until day 10 (full-blown DSS-induced colitis). Mice were >10 weeks of age. For evaluation of colitis induction and development, mice were analyzed on days 2, 4, 6, and 10 (Supplementary Figure S1). Controls received pure drinking water during this time course.

Transfer colitis was induced in 8-week-old *Rag* ^*1tm1Mom*^ mice by i. p. injection of 4 × 10^5^ CD4^+^CD45RB^hi^ T cells isolated from spleens of WT mice using a protocol according to Maschmeyer *et al.* (Maschmeyer et al., 2021). Control animals received only phosphate buffered saline (PBS, Thermo Fisher Scientific). For full-blown transfer colitis, mice were analyzed on day 40. For the evaluation of inflammation during induction, development and chronicity, mice were analyzed on days 14, 21, 28 and 40 (Supplementary Figure S1). Mice were controlled regularly for clinical signs of colitis and electively sacrificed when meeting humane endpoints. Stool samples were scored as described elsewhere (Erben et al., 2015).

### Magnetic Resonance Imaging

VSOPs doped with europium (Eu-VSOPs, further simplified as VSOPs) were produced by the Department of Radiology - Experimental Radiology (Charité - Universitätsmedizin Berlin) as described previously (de Schellenberger et al., 2017). Colitic and control mice were anesthetized via a mask (2% isoflurane in a mixture of 30% O_2_ and 70% N_2_O; Baxter). Mice were placed on a warming pad, fixed on an animal holder and respiration was continuously monitored (Small Animal Instruments Inc., Stony Brook, NY, United States). After acquisition of MRI scans (pre-MRI), the animals received 0.03 mmol Eu-VSOP per kg body weight via the tail vein. After 90 min, a second set of MRI scans was acquired (post-MRI).

MRI was performed in a preclinical 7 T MRI scanner (BioSpec, Bruker, Ettlingen, Germany) running with Paravision 6.0.1 software. All scans were acquired with a 35-mm diameter 1H-RF quadrature volume coil (RAPID Biomedical, Würzburg, Germany). The protocol consisted of a T_2_-weighted 2D RARE scan with field of view (FOV) = 33 × 33 mm^2^, 60 contiguous 0.75 mm thick axial slices, matrix dimension (MTX) = 220 × 216 zero filled to 220 × 220 (achieved using 1.02 partial fourier acceleration), echo train length (ETL) = 8, echo spacing (ΔTE) = 8 ms, effective echo time (TE) = 24 ms, repetition time (TR) = 4,800 ms, readout bandwidth (BW) = 50 kHz, total acquisition time (TA) = 6:29 min and a T_2_^*^-weighted 2D FLASH scan with identical geometry, MTX = 120 × 148 zerofilled to 220 × 220, TE = 3 ms (achieved using 1.49 partial fourier acceleration), TR = 605 ms, BW = 44,643 Hz, TA = 2:59 min. All MRI protocols worked with fat suppression and flipback.

### MRI Analysis

Image analysis was done with ImageJ (Java version 1.6.0_20). Six sub-sequential images (further referred to position 1–6) obtained from axial T_2_^*^-weighted (T_2_^*^w) scans were analyzed in a blinded manner by two researchers (Supplementary Figure S1). A region of interest (ROI) was drawn around the colon wall by fitting a circle around the outer and inner colon wall. When the colon did not show any lumen in the respective image, only one circle was drawn around the colon wall. Signal intensity of the ROI was measured with ImageJ. In MRI image-slices one pixel equals 0.0225 mm^2^. In the ROI of the colon tissue 195.393 ± 69.985 pixel were analyzed. Pixel number was calculated by dividing the area of ROI_Colon_ with pixel size of one pixel (Area_Colon_/0.0225 mm^2^). Contrast-to-noise ratio (CNR) was calculated by subtraction of the signal-to-noise in the colon (SNR_colon_) from the SNR_muscle_ (CNR = SNR_colon_- SNR_muscle_). The muscle was chosen as reference tissue in this study. SNR was calculated by dividing the signal of the tissue of interest by the standard deviation (SD) of the background signal (SNR_tissue of interest_ = Signal_tissue of interest_/SD_Noise_). The background signal was measured by drawing a rectangular area in the upper part of the images outside of the body.

### Histopathology

Directly after MRI analysis, mice were sacrificed by cervical dislocation under anesthesia. The large and small intestine as well as the liver were removed and were fixed in 10% formalin (SAV Liquid Production) overnight at room temperature. Formalin-fixed tissue was embedded in paraffin (Histosec, Merck) and paraffin blocks were prepared. Sections were freshly cut for histochemistry and immunohistochemistry (1–2 µm). For imaging mass cytometry (IMC), 4 µm thick paraffin sections were cut from a tissue microarray prepared from paraffin blocks of colon and liver tissue.

For the evaluation of histomorphology (scoring), paraffin sections were dewaxed, stained with hematoxylin and eosin (both Merck) and coverslipped with Histokitt (Roth). The scoring schemes for intestinal inflammation are described elsewhere (Erben et al., 2014). Hepatitis was scored according to Siegmund *et al.* (Siegmund et al., 2002). Additionally, hematoxylin and eosin-stained sections were used for the designation of the location of punches for the tissue microarray.

For immunohistochemistry, paraffin sections were dewaxed and subjected to heat-induced epitope retrieval prior to incubation with anti-CSPG4 (polyclonal rabbit, Sigma SAB4301658) at 4°C overnight. For detection, the EnVision + System-HRP Labelled Polymer Anti-Rabbit (Agilent) was used. HRP was visualized with diaminobenzidine (Agilent) as chromogen. After inactivation of proteins and enzymes, sections were incubated with the biotinylated HA binding protein (Millipore #385911) for 30 min at room temperature followed by incubation with alkaline phosphatase-labelled streptavidin and RED as chromogen (both Agilent). Nuclei were counterstained with hematoxylin (Merck) and slides coverslipped with glycerol gelatine (Merck). Stained sections were evaluated by an AxioImager Z1 (Carl Zeiss Microscopy GmbH). Controls were performed by omitting the primary antibodies. Using the Vectra3 microscopy system (Akoya Biosciences), multispectral images (5 high power fields (HPF) per colon section) were acquired. HPF were chosen manually in a blinded manner. The inform software (version 2.4.7174.15475) allows for spectral unmixing of the images. Unmixed images showing CSPG4, HA, and nuclear staining were analyzed separately in ImageJ (Java version 1.6.0_20) using the plugin “IHC Toolbox”. The positively stained area was measured in arbitrary unit and the mean from five HPF was calculated per colon.

### Imaging Mass Cytometry

IMC was performed as described elsewhere (Lehmann et al., 2021). Briefly, paraffin sections of tissue microarrays were dewaxed prior to heat-induced antigen retrieval at pH 6.0 followed by a blocking step in 3% BSA (Albumin Bovine Fraction V, Serva, Heidelberg, Germany) in antibody diluent (Agilent, Santa Clara, United States). Sections were incubated overnight at 4 °C with a cocktail of metal-conjugated antibodies (Table 1) prepared in 0.5% BSA-containing antibody diluent (Agilent). Pre-labeled antibodies were purchased from Fluidigm (San Francisco, United States). Unlabeled antibodies were purchased in carrier-free buffer as indicated in Table 1 and labeled using MaxPar Antibody X8 conjugation kits (Fluidigm) according to the manufacturer’s instructions and reconstituted in PBS-based antibody stabilizer (CANDOR Bioscience, Wangen, Germany) at 0.5 mg/ml. Nuclei were stained with CELL-ID Intercalator-Ir (1:400, Fluidigm) in antibody diluent (Agilent). Slides were rinsed, air-dried, and stored at room temperature until measurement.

**TABLE 1:** Antibody cocktail for imaging mass cytometry.

Isotope	Antigen	Clone	Company	Dilution
169 Tm	CD45	MRC OX-1	Abcam	1:5,000
170Er	CD3ε	D4V8L	Fluidigm	1:200
156Gd	CD4	D7D2Z	CST	1:100
173 Yb	CD8α	4SM15	ThermoFisher Scientific	1:100
148Nd	CD44	E7K2Y	CST	1:1,000
166Er	B220	RA3-6B2	ThermoFisher Scientific	1:8,000
168Er	CD138	281–2	BioLegend	1:10,000
145Nd	F4/80	EPR22059-270	Abcam	1:200
163Dy	CD68	Polyclonal	Abcam	1:400
150Nd	Ly6G	1A8	BioLegend	1:200
149Sm	CD11b	EPR1344	Fluidigm	1:400
154Sm	CD11c	Polyclonal	Fluidigm	1:200
146Nd	CD56	E7X9M	CST	1:200
172 Yb	Vimentin	EPR3776	Abcam	1:15,000
174 Yb	αSMA	Polyclonal	Abcam	1:15,000
158Gd	E-Cadherin	4A2	Abcam	1:5,000
143Nd	EpCAM	E6V8Y	CST	1:5,000
167Er	beta-catenin	6B3	CST	1:10,000
175Lu	CD103	BP6	Abcam	1:200
164Dy	CD31	D8V9E	CST	1:5,000
147Sm	Ki67	16A8	BioLegend	1:400
176 Yb	clCasp3	Asp175	CST	1:100
151Eu	EuVSOP			
153Eu	EuVSOP			
191Ir	Cell-ID Intercalator		Fluidigm	
193Ir	Cell-ID Intercalator		Fluidigm	

For IMC data acquisition, a CyTOF2/upgraded to Helios specifications coupled to a Hyperion Tissue Imager (Fluidigm) was used employing CyTOF software (v6.5.236). The instrument was tuned according to the manufacturer´s instructions prior to loading the dried slide into the imaging module. Optimal laser power was determined for colon and for liver to ensure complete ablation of the tissues. Laser ablation was performed at a resolution of 1 µm and a frequency of 200 Hz. Data were stored as mcd- and txt-files.

Single-cell analysis was done as published before (Lehmann et al., 2021). The data was not transformed prior to analysis in HistoCAT and the HistoCAT gating function was used to gate out unspecific cell debris in the intestinal lumen of the DSS-induced colitis samples. For cell clustering, a dimensionality reduction tSNE algorithm was used to visualize all single cell data excluding the europium, the β-catenin and the E-Cadherin channels. For cluster analysis the Phenograph function of HistoCAT served to cluster cells according to their marker expression with k nearest neighbor = 100 for colon and k nearest neighbor = 80 for liver samples analysis.

Cell count quantification was done with Cytobank software.

### Laser Ablation Inductively Coupled Plasma Mass Spectrometry (LA-ICP-MS)

Paraffin sections of colon tissue were cut from a tissue microarray. Laser ablation inductively coupled plasma mass spectrometry (LA-ICP-MS) analysis was performed on a commercial LA system (NWR-213, ESI, Bozeman, MT, United States) equipped with a two-volume sample chamber coupled to a sector field ICP-MS (Element XR, Thermo Fisher Scientific, Bremen, Germany). Argon was added as sample gas, 0.690 L/min to the helium carrier gas, 1 L/min, through a Y-piece. The instrument performance was tuned daily for signal intensity and stability (RSD <5%), as well as low oxide rate (ThO/Th < 1%) using a glass slide. Correction of instrumental drift and quantification was carried out by ablation of matrix matched agarose gels cast on glass slides (0–55 fg/pixel). Overview of LA-ICP-MS parameters are summarized in Supplementary Table S1.

### Statistical Analysis

Group comparisons were carried out using Mann-Whitney test with *p* ≤ 0.05 defining the statistical significance. Results are displayed as mean ± SD using GraphPad Prism (Version 6).

## Results

### VSOPs Induce Strong Signal Loss in Colon MRI of Mice With DSS-Induced Colitis but Not of Mice With Transfer Colitis

**FIGURE 1:**
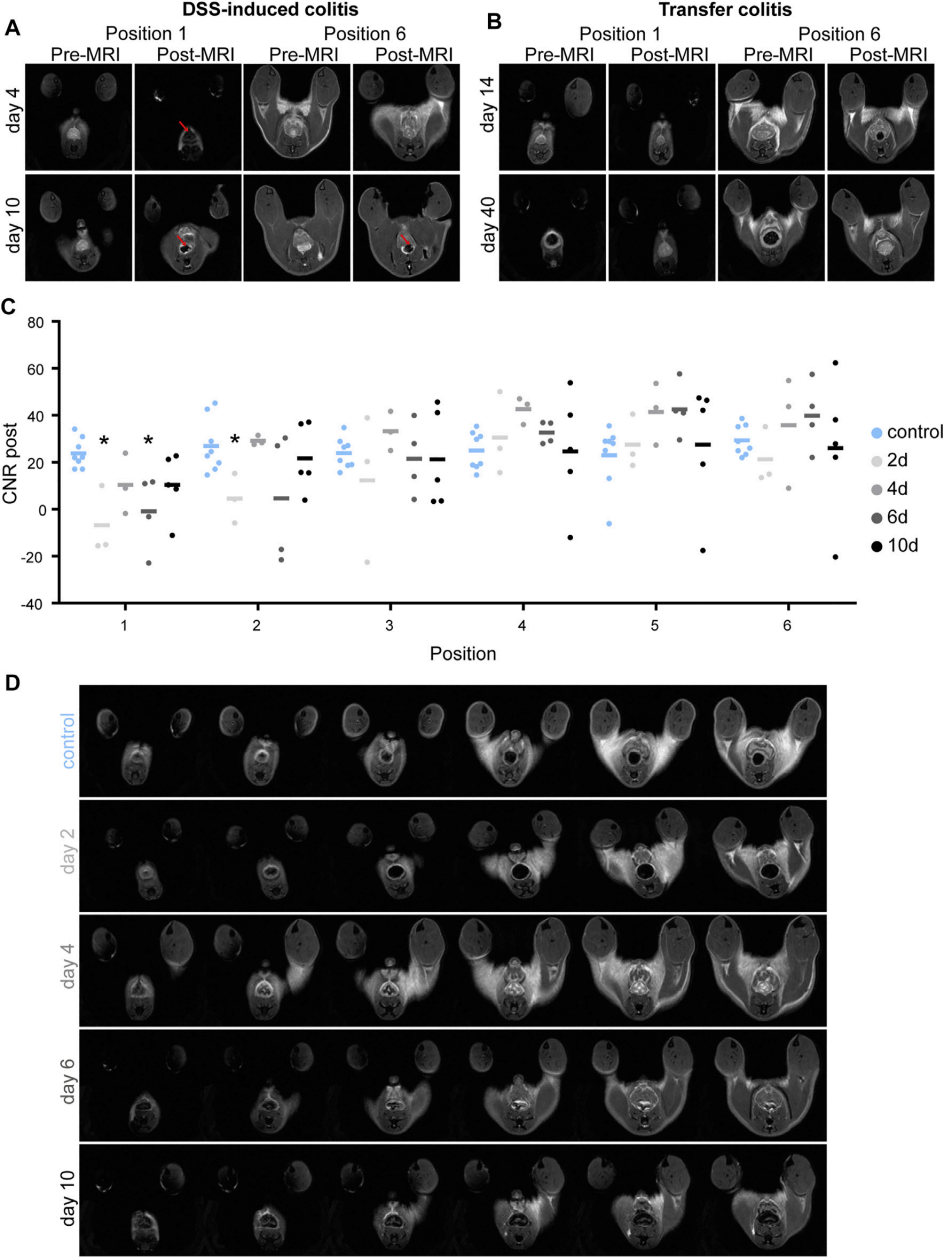
Accumulation of VSOP-induced signal loss in the colon of DSS-induced colitis mice. **(A)** Representative pre- and post-colon MRI images of mice with DSS-induced colitis at days 4 and 10, respectively. Upper row shows images of pre- and post-MRI scans of a mouse with DSS-induced colitis on day 4 in position 1 and 6, respectively. Lower row indicates images of pre- and post-MRI scans of a mouse with DSS-induced colitis on day 10 in position 1 and 6, respectively. Red arrows point to areas of signal loss indicating VSOP accumulation. **(B)** Representative colon MRI images of mice with transfer colitis at days 14 and 40. Upper row presents images of pre- and post-MRI scans of a mouse on day 14 after transfer colitis induction in position 1 and 6, respectively. Lower row indicates images of pre- and post-MRI scans of a transfer colitis mouse on day 40 in position one and 6. There is no signal loss discernible. **(C)** Graphic summary of contrast to noise ratio (CNR) in colon wall of position one to six, at days 2 (*n* = 3), 4 (*n* = 3), 6 (*n* = 4), and 10 (*n* = 5). The CNR in colon wall of healthy control animals is represented in blue for every position (*n* = 7). Statistical significance was tested with Mann-Whitney test (non-parametric *t*-test) with *p* ≤ 0.05 defining the statistical significance that is marked with ^∗^. **(D)** Representative sub-sequential axial post-MRI images from one animal of each group.

In a T_2_*-weighted (T_2_*w) MRI scan, VSOP accumulation induces a signal loss and areas appear black. Colon MRI of mice with DSS-induced colitis and with VSOP injection showed signal loss in potentially inflamed sites of the colon. Figure 1A displays representative colon MR images at early (day 4) and late disease stages (day 10). The accumulation of VSOPs and signal loss in post-MRI colon scans could not be observed in colon wall of the healthy control group indicating that VSOP accumulation is reflecting colon wall inflammation (Supplementary Figure S2). In the DSS-induced colitis model, inflammation starts in the distal colon and spreads continuously to the proximal colon. The post-MRI scans in this model nicely reflected this pattern. In animals at earlier time points (day 4) signal loss was present in the first but not in the later more proximal positions of post-MRI images, from position 5 onwards. With disease progression, the inflammation spread to the descending colon, this is reflected in post-MRI scans of animals with severe inflammation (days 6 and 10). Here, signal loss was detected in more proximal positions as well (Figure 1A, day 10). Strikingly, in mice with transfer colitis, VSOP accumulation and signal loss was not detectable in any of the MRI scans (Figure 1B).

Signal loss in the colon wall of all six positions was further quantified by calculation of contrast-to-noise ratio (CNR) and results are summarized in Figure 1C. Lower CNR, indicated more signal loss in the colon tissue. For all time points of DSS-treatment the CNR in position 1, the most distal part of the colon, showed a tendency to be lower than the CNR in the healthy control group. Decrease of CNR at 2 days after DSS treatment is significantly lower in position 1 (*p* = 0.0121) and position 2 (*p* = 0.0242) and for 6 days of DSS treatment in position 1 (*p* = 0.004), when compared to control. In animals with less inflammation, the CNR increased again, comparable to control value, latest at position 4, the more proximal part of the colon. This indicates non-inflamed tissue in the proximal position as already seen by eye in the MRI scans (Figure 1D). In severe DSS-induced colitis (day 10), the CNR was partially lower than control values, however failed to be significant, indicating spreading of the inflammation to the more proximal colon. This is in line with model characteristics as DSS-induced colitis is most pronounced in the distal colon and spreads continuously to the proximal colon.

**FIGURE 2:**
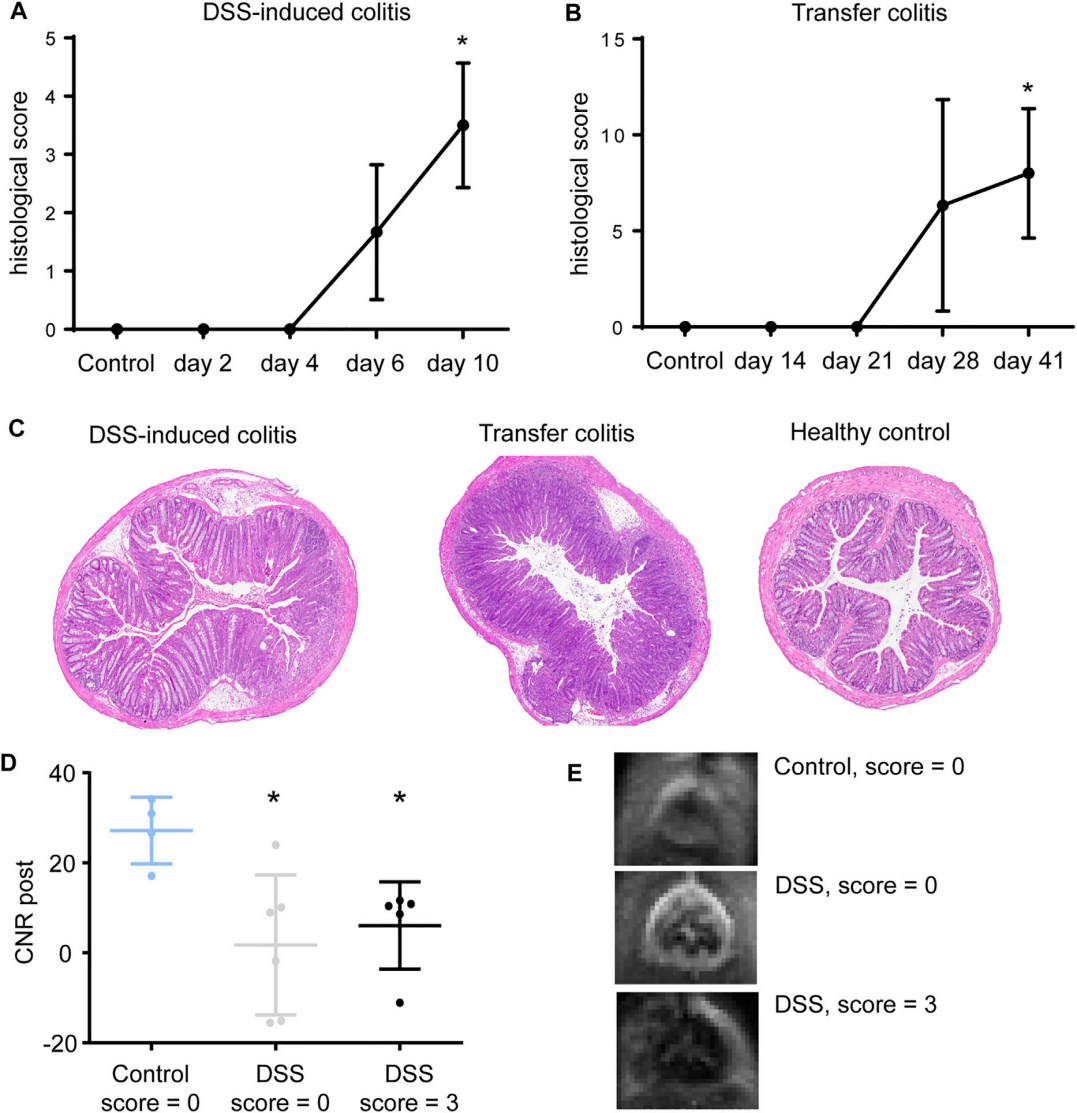
CNR decrease in histologically non-inflamed colon tissue. **(A)** Mice with DSS-induced colitis presented with an increased score starting at day 4 (mean ± SD, *n* = 2–6) when compared to controls (*n* = 3) Statistical significance was tested with Mann-Whitney test (non-parametric *t*-test) with *p* ≤ 0.05 defining the statistical significance that is marked with *. **(B)** Transfer colitis mice show increase in histopathological inflammation score starting at day 21 (mean ± SD, *n* = 2–4) compared to controls (*n* = 4). Statistical significance was tested with Mann-Whitney test (non-parametric *t*-test) with *p* ≤ 0.05 defining the statistical significance that is marked with *. **(C)** Representative images of H&E-stained colon sections of DSS-induced colitis mouse at day 10 with manifestation of severe ulceration and tissue changes, transfer colitis mouse showing severe crypt hyperplasia and immune cell infiltration and healthy control mouse. **(D)** CNR in position one of colon MRI of DSS-treated animals with histological score of 0 and 3 with respective control (mean ± SD, *n* = 5–6). Animals showed a significantly lower CNR (CNR post) when scored with 0 (*p* = 0.019) or 3 (*p* = 0.0159) when compared to CNR of the healthy control group. Statistical significance was tested with Mann-Whitney test (non-parametric *t*-test) with *p* ≤ 0.05 defining the statistical significance that is marked with ^∗^. **(E)** Representative images at position one of colon regions (distal colon) where no signal loss was observed in the colon wall of healthy animals, but severe signal loss in DSS-induced colitis mice with histology score of 0 or 3.

However, scoring of colon tissue showed successful manifestation of inflammation in both, DSS-induced (Figure 2A) and transfer colitis (Figure 2B). Strikingly, DSS-treated animals with a histological score of 0 already presented a signal loss in the colon in the MRI scans, indicating early VSOP accumulation. When comparing DSS-treated animals with a score of 0 with healthy control animals also scored 0, the respective CNR in the first position of the MRI scans is significantly decreased (*p* = 0.019). Animals with a histological score of three showed even lower CNR compared to both groups (*p* = 0.0159) (Figures 2D, E). This underlines a more sensitive detection of potential ECM-associated tissue changes in the colon by MRI, as a signal loss was detectable in the colon of animals that histopathologically showed no overt inflammation.

### ECM Composition Changes During Colitis Course and Differs in DSS-Induced Colitis and Transfer Colitis

Inflammation in both models has knowingly different causes and manifestations bringing along different tissue compositions. Leading to the question what difference in the tissue composition influences the binding and accumulation of VSOPs in the DSS-induced colitis compared to the missing accumulation in transfer colitis. To understand the distinct binding-capacity of VSOP in the different colitis models, the ECM composition was elucidated by HPLC, as described in Volpi et al. (2014) and Biskup et al. (2021), and histopathology.

**FIGURE 3:**
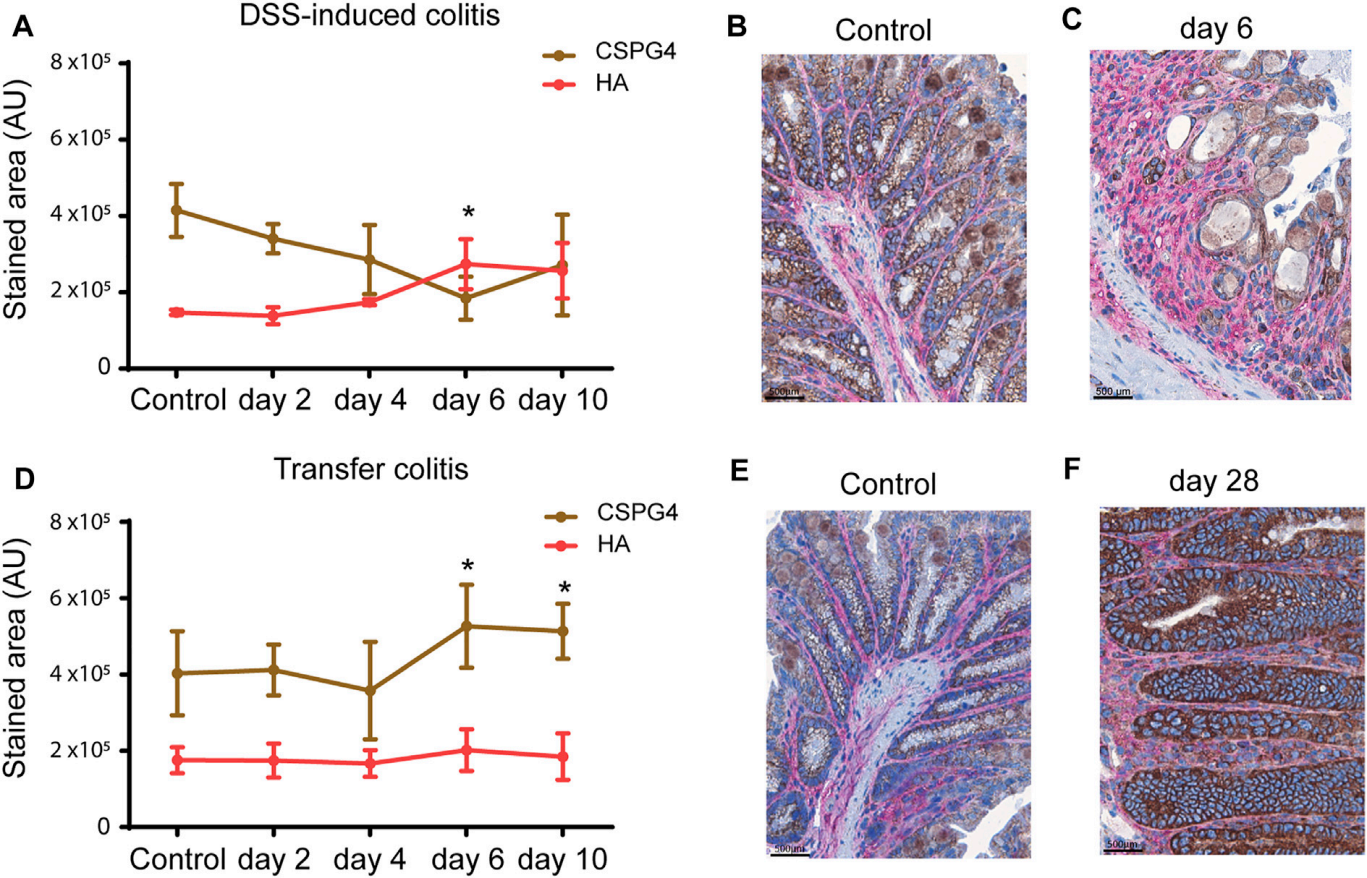
HA and chondroitin sulfate are differently expressed in health and intestinal inflammation as well as in DSS-induced colitis and transfer colitis. **(A)** Expression of HA (red) and CSPG4 (brown) quantified as positive stained area (mean AU ± SD) on day 2 (*n* = 5), day 4 (*n* = 4), day 6 (*n* = 9) and day 10 (*n* = 12) show an increase in HA and decrease in CPSG4 compared to controls (*n* = 3). Representative images of colon sections from mice with DSS-induced colitis immunohistochemically stained for HA (red) and CSPG4 (brown) shown for healthy **(B)** and inflamed colon **(C)**. **(D)** Expression of HA and CSPG4 quantified as positive stained area (mean AU ± SD) on day 14 (*n* = 3), day 21 (*n* = 5), day 28 (*n* = 6) and day 41 (*n* = 9) show no changes in HA content but an increase in CSPG4 compared to controls (*n* = 24). Representative images of colon sections from mice with transfer colitis immunohistochemically stained for HA (red) and CSPG4 (brown) shown for healthy **(E)** and inflamed colon **(F)**.

Hyaluronic acid (HA) and chondroitin sulfate (CS) are two of the major components of the ECM. DSS treatment led to an increased content of HA compared to controls detected by HPLC. While chondroitin sulfate 4S (CS-4S) decreased with inflammation severity (Supplementary Figure S3A), the opposite occurred in mice with transfer colitis: the amount of HA in colon tissue strongly decreased compared to control mice, whereas the amount of CS-4S increased (Supplementary Figure S3B). HPLC analysis indicated that the colon ECM composition differed in both models. For spatial allocation, HA and CS proteoglycan 4 (CSPG4) were detected by immunohistochemistry. Quantification of the immunohistochemically stained tissue areas supported the HPLC data and confirmed an increase of HA and a decrease of CSPG4 in colon tissue of mice with DSS-induced colitis (Figure 3A). In healthy control mice HA was expressed in the mucosa and submucosa, CSPG4 was expressed by epithelial and goblet cells in the mucosa (Figure 3B and Supplementary Figure S4A). After 2 days of DSS treatment, the expression of HA in mucosa and submucosa was slightly increased, whereas CSPG4 expression was comparable to baseline (Supplementary Figure S4B). By day 4, the submucosa is edematous, but the expression level of HA remained slightly increased. The HA expression in the mucosa was again increased. The expression of CSPG4 in the mucosa was still unchanged, whereas sporadic CSPG4-positive cells were present in the submucosa (Supplementary Figure 4C). Highly inflamed areas of the mucosa (arrows) expressed high amounts of HA. Additionally, the HA expression was increased within the edematous submucosa. Some scattered CSPG4-expressing cells could be found in the submucosa. The amount of CSPG4 though was decreasing in the mucosa due to loss of crypts and goblet cells (Figure 3C and Supplementary Figure S4D). The inflammation further deteriorated when switching from DSS to drinking water. After 2 days of drinking water, the highly inflamed mucosa showed high expression of HA and low expression of CSPG4 (Supplementary Figure S4E). The expression of HA was still high in the submucosa with increased numbers of cells expressing CSPG4. Ulcerations were nearly devoid of both HA and CSPG4. After 4 days of drinking water (day 10 of the experiment), the expression level of HA remains unaltered, whereas CSPG4 expression was increased in regenerating crypts (Supplementary Figure S4F). Remarkably, in transfer colitis mice no changes in HA content in colon tissue occurred. Similar to the HPLC analysis, CSPG4 was significantly increased at day 28 and 40 (Figure 3D). In colon tissue of healthy controls (*Rag* ^*1tm1Mom*^ mice), HA localized as a thin band in the submucosa and between the crypts, whereas CSPG4 was restricted to goblet cells within the crypts in both models (Figure 3E and Supplementary Figure S5A). After 14 days of T-cell transfer, the expression pattern is unchanged with a minor increase in HA and CSPG4 in the mucosa (Supplementary Figure S5B). By day 21, both HA and CSPG4 remain slightly increased and on day 28, CSPG4 expression was increased in the mucosa. HA was slightly increased within the submucosa but not mucosa (Figure 3F, Supplementary Figures 5C, D). At the end of the experiment (day 40), expression level and amount of CSPG4 remained high in the mucosa (Supplementary Figure S5E). The expression of HA was also unaltered and still increased in the submucosa.

### In Liver Tissue VSOPs Are Associated With Macrophages Within Sinusoids

Due to i. v. application of the VSOPs, leukocytes and endothelial cells are the first cells getting in contact with the VSOPs. Under basal conditions, the liver is the organ with the highest blood supply followed by kidneys, muscles, and brain. Blood is supplied to the liver via the hepatic artery and the portal vein. As mice with severe transfer colitis additionally have been described to develop a bystander hepatitis (Powrie et al., 1993) liver tissue sections were analyzed by IMC for localization of i. v. applied VSOPs.

**FIGURE 4:**
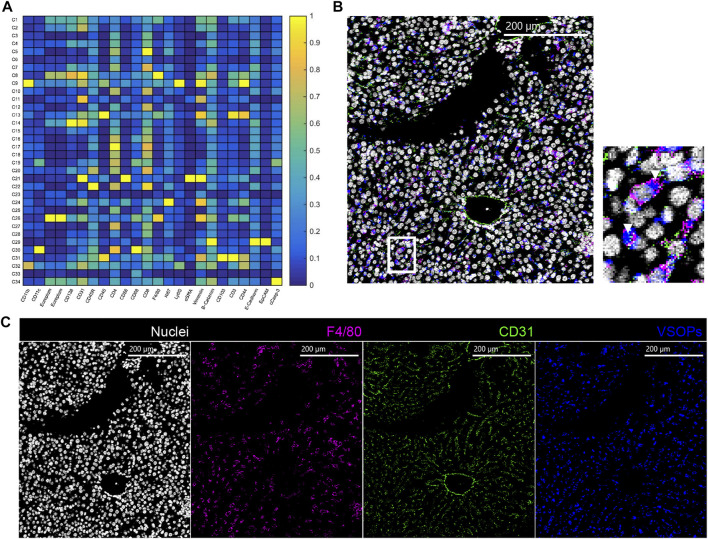
VSOP in the liver are associated with sinusoidal macrophages. **(A)** Heatmap of liver tissue analysis shows co-localization of VSOPs with cluster eight und cluster 26. In representative IMC image of a transfer colitis mouse at day 40 and injected with VSOPs, nuclei are presented in white, VSOPs in blue, CD31 in green and F4/80 in magenta in a merged image and a zoomed in region of the liver with white arrow heads pointing at VSOP accumulation **(B)**. Individual images for nuclei, F4/80, CD31 and VSOPs **(C)**.

IMC analysis of liver tissue from the transfer colitis model and healthy controls revealed high accumulation of VSOPs in liver tissue independent of treatment. VSOPs mainly co-localize with F4/80^+^ macrophages within the sinusoids (Figure 4A). In liver tissue from healthy animals VSOPs accumulate within F4/80^+^ macrophages and CD31^+^ endothelial cells (Supplementary Figure S10B). This is also true for liver tissues from mice with transfer colitis exhibiting bystander hepatitis, where VSOPs do not accumulate in the inflammatory infiltrate (Figures 4B, C, Supplementary Figure S11B; Supplementary Figure S12B).

### VSOP Signal in Colon Tissue Is Associated With Vessels and Macrophages

**FIGURE 5:**
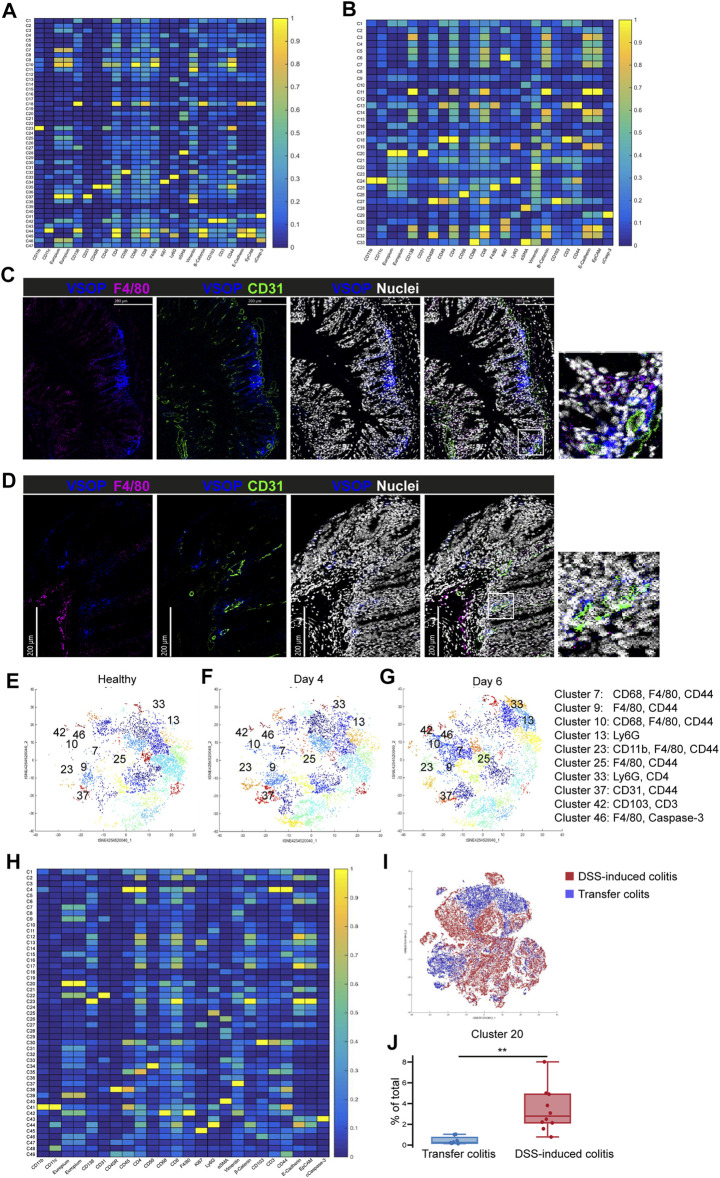
VSOPs accumulate in vessels and macrophages in colon tissue of DSS-induced colitis mice and to a lower extent in transfer colitis mice. Heatmap summarizes computational analysis of colon tissue from mice with DSS-induced colitis. VSOPs accumulate within CD31^+^ endothelial cells as well as with CD45^+^ hematopoietic cells, specifically with CD68^+^, F4/80^+^ macrophages expressing CD44, CD3^+^ T cells expressing CD103^+^, CD11b^+^ macrophages in DSS-induced colitis **(A)** In transfer colitis, VSOPs most prominently cluster with CD31^+^ endothelial cells (cluster 20) and to lower extent with F4/80^+^ macrophages (cluster 25) and CD11b^+^, CD11c^+^ and Ly6G^+^ macrophages (cluster 24) **(B)**. Representative IMC images of a DSS-induced colitis mouse at day 6 **(C)** and a transfer colitis mouse at day 40 **(D)** injected with VSOPs, with F4/80 in magenta, CD31 in green, VSOPs in blue and nuclei in white. Phenograph clustering of DSS-induced colitis colon samples with two mice per group for healthy control **(E)**, 4 days of DSS treatment **(F)** and 6 days of DSS treatment **(G)** (*n* = 2) shows, that with increasing colon inflammation, the VSOPs clusters become more prevalent. The clusters containing VSOPs are labeled with respective numbers (markers within the clusters are listed in the legend on the right-hand side). Heatmap summarizes an additional computational analysis with colon tissue from DSS-induced colitis model and transfer colitis model used together for tSNE algorithm and phonograph clustering **(H)**. Visualization of cluster distribution with all DSS-induced colitis samples in red and transfer colitis samples in blue **(I)**. The relative amount of cells in cluster 20 (containing VSOPs, and macrophages (F4/80^+^, CD68^+^) and CD44 expressing cells) is significantly higher in DSS-induced colitis compared to transfer colitis (*p* = 0.001592). Statistical significance was tested with Student’s t-test with *p* ≤ 0.05 defining the statistical significance that is marked with ^∗^. **(J)**.

**FIGURE 6:**
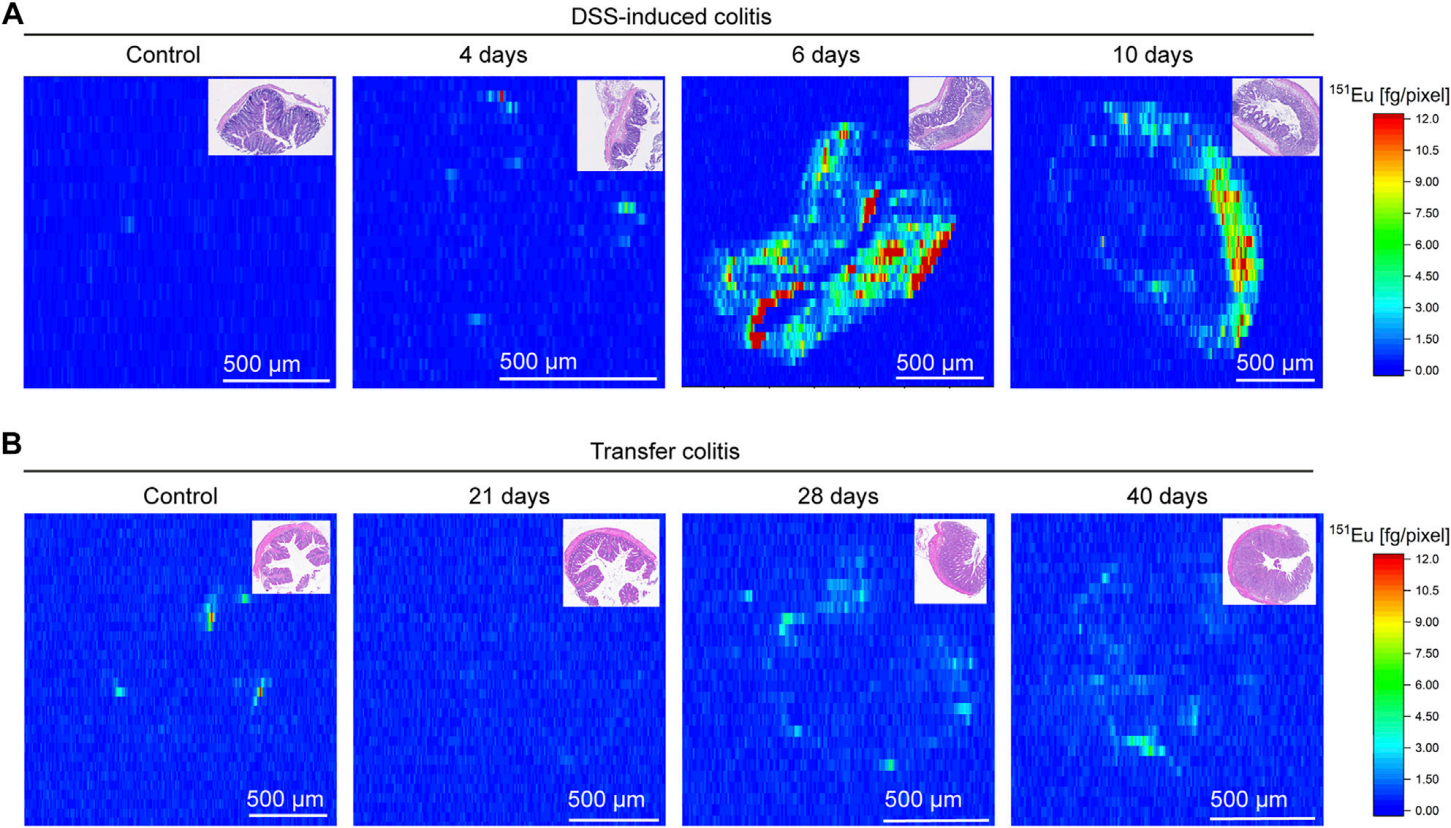
Europium (Eu) content measured with laser ablation inductively coupled mass spectrometry (LA-ICP-MS) is higher in colon tissue of DSS-induced colitis as in transfer colitis. Representative elemental maps of Eu in DSS-induced colitis **(A)** at day 4, 6, and 10 with respective control and respective H&E staining of measured sample in the upper right corner. The scale bar refers to the amount of Eu in respective pixel in fg/pixel. In transfer colitis **(B)** the representative elemental maps for different time points (day 21, 28 and 40) and healthy control generated with LA-ICP-MS underline lower abundance of Eu and therefore VSOPs. The H&E staining of measured tissue is shown in the upper right corner.

Analysis of IMC analyses of colon tissue from mice with DSS-induced colitis and transfer colitis revealed that VSOPs co-localized with CD31^+^ endothelial cells in both models (Figures 5A, B). However, in DSS-induced colitis VSOPs further strongly co-localized with CD68, F4/80 double positive macrophages expressing CD44 (Figure 5A). In transfer colitis mice, VSOPs co-localize to F4/80, CD68 double positive macrophages to a much lesser extent. Additionally, a low signal of VSOPs is present in macrophages expressing Ly6G, CD11b and CD11c (Figure 5B). Representative IMC images of colon tissue from animals with severe inflammation underlines a higher abundance of VSOPs in the colon from DSS-induced colitis (Figure 5C) when comparing to VSOPs abundance in colon from a transfer colitis mouse (Figure 5D). VSOPs reached the colon tissue in transfer colitis during severe inflammation, but the amount strongly decreased when the tissue was less inflamed (Supplementary Figure S11A; Supplementary Figure S12A). However, the amount of accumulated VSOPs during severe inflammation in the transfer colitis model was not sufficient to be visualized by MRI. In contrast, representative IMC images of day 4 and 6 of DSS-induced colitis animals revealed accumulation of VSOPs within colon tissue during mild and moderate inflammation (Supplementary Figures S7, 8), whereas in healthy colon tissue VSOPs could only be detected within the vessels and no accumulation was detectable within the tissue (Supplementary Figure S6). Phenograph clustering of colon tissue from DSS-induced colitis at day 4 and 6 as well as the respective control, further showed a lack of VSOPs clusters, in healthy mice (Figure 5E), whereas in the less inflamed group (Figure 5F) the VSOPs clusters were less abundant, when compared to severely inflamed tissue (Figure 5G). New tSNE clustering of all DSS-induced colitis and transfer colitis samples (Figure 5H) underlined that the different mouse models contain different types of cells in colon tissue (Figure 5I). This is emphasized by a significant lower abundance (*p* = 0.001592) of total amount of VSOPs containing F4/80^+^, CD68^+^ macrophages, expressing CD44 in transfer colitis (Figure 5J). With laser ablation inductively coupled plasma mass spectrometry (LA-ICP-MS) the presence of VSOPs in colon tissue confirmed the observations with IMC (Figure 6). For DSS-induced colitis the presence of europium in the colon increased with inflammation, while the measured content was low in respective control (Figure 6A). In colon tissue of transfer colitis animals the europium content analyzed with LA-ICP-MS was constantly low, independent of disease stage (Figure 6B).

## Discussion

In IBD, early diagnosis and disease surveillance are of importance to limit long-term complications. The current gold standard is endoscopy/colonoscopy and either ultrasound or MRI together with histological evaluation of biopsies (Gomollon et al., 2017; Magro et al., 2017). However, follow-up measures that allow for early detection of structural changes are currently limited. MRI has great potential to be a non-invasive tool for this. Presently, MRI represents the standard for assessing small intestinal inflammation in CD (Gomollon et al., 2017). Preclinical studies in mice successfully applied non-contrast agent enhanced MRI to assess colon inflammation by the estimation of colon wall thickness (Beltzer et al., 2016; Bianchi et al., 2016; Biton et al., 2018). Some studies suggested to additionally reduce bowel movement by medication and fasting to get higher quality MRI scan. Further, Fluorinert, a perifluorinated liquid that does not produce a proton MR signal, can be administered to create homologous background in colon lumen to reduce unspecific signal in MRI images (Ileva et al., 2014). In the study presented herein, VSOPs were applied to increase sensitivity in detecting inflammatory regions and changes in the extracellular matrix in colon tissue. VSOPs assist a sensitive detection of inflammation and ECM changes in the colon tissue already at the presence of a minimal colitis, without the need to include background reducing agents for the lumen, fasting of animals or medication to reduce bowel movement. The main advantage of MRI is the imaging of the whole colon by possible simultaneous detection of extraintestinal manifestations. This is neither possible with endoscopy/colonoscopy nor with histopathology. In endo- and colonoscopy, the intestine is only visible from the luminal side and not every part of the intestine can be reached by the endoscope. Biopsies taken during endo-/colonoscopy are usually taken from the (sub)mucosa. In contrast to MRI, no full-thickness bowel wall examination is feasible in routine examinations. Full-thickness bowel wall examination by MRI is reflected by the decrease of the CNR in colon wall tissue of DSS-induced colitis animals during mild colitis (day 2 and day 4), when the tissue appeared histomorphologically normal. Nevertheless, the limitations of transferring a small animal set up to a clinical set up for humans needs to be considered (Herrmann et al., 2012; Hoyer et al., 2014). When sampling of colon tissue for histological scoring, the location where the biopsy is taken plays an important role. This could strongly limit a precise scoring. However, in this study the samples were taken distally, exactly where the highest accumulation of VSOPs was detected in parallel to the maximum of inflammation in the DSS-induced colitis model. The applied scoring system was further specified for each of the mouse models and thus provided a very accurate evaluation of distinct changes within the tissue (Erben et al., 2014). This histomorphological scoring system takes into account mild mucosal cell infiltration. However, small changes like alterations of cell types, changes of components in the ECM and very small cell infiltrations will not be reported. Therefore, VSOPs accumulation within early onset inflamed colon tissue can be explained with very early changes within the tissue. These changes seem to happen before immune cell infiltration. Highlighting the sensitivity of contrast-enhanced MRI. Accumulation of VSOPs in healthy colon tissue can be ruled out as shown by IMC, underlining the specificity of the VSOPs. Multiple iron oxide based contrast agents were approved by the FDA (Food and Drug Administration) starting in 1996. However, most of them were canceled again due to safety concerns (Dulinska-Litewka et al., 2019). The only current FDA approved iron oxide based contrast agent is ferumoxytol (Thakor et al., 2016). Most contrast agents that appeared on the market were mainly used for MRI of the liver or the spleen, as due to their rather large particle size they are taken up by cells of the mononuclear phagocytic system (Bernd et al., 2009; Wang 2011). VSOPs however, have a smaller hydrodynamic diameter (7 nm), thus, up-take should not be limited to cells of the mononuclear phagocytic system (Bernd et al., 2009). IMC computational analysis of the liver from mice with transfer colitis suggested that VSOPs were taken up mainly by the Kupffer cells in the liver (F4/80^+^, CD68^+^ and Ly6G^−^). Nevertheless, VSOPs accumulation increased with further development of inflammation in the colon of DSS-induced colitis and was mainly occurring in macrophages. Strikingly, in the transfer colitis model no VSOP-induced signal loss in early inflammation and at more chronic time points in severely inflamed colon tissue was detected with MRI. IMC and LA-ICP-MS analysis of colon tissue from transfer colitis mice demonstrated the presence of VSOPs though to a lesser extent than in inflamed colon tissue of DSS-induced colitis mice. This low amount of VSOPs was not able to induce a detectable signal loss in MRI. Furthermore, the IMC data displayed a lower amount of CD44-expressing macrophages in the colon of transfer colitis mice. The transmembrane receptor CD44 is commonly known to be expressed on macrophages and is mainly involved in the binding of HA (Vachon et al., 2007). Additionally, CD44 mediates interaction of macrophages with the endothelium (Kennel et al., 1993). HA and the enzymes responsible for its synthesis, hyaluronan synthase -1, -2 and -3 (HAS1, HAS2 and HAS3), play a central role in driving gut inflammation (Kessler et al., 2015). In DSS-treated mice, the deposition of HA within blood vessels of colon tissue can already be observed at day 3 of DSS treatment. This deposition then induces an immune cell influx into the damaged gut tissue (Kessler et al., 2008). As CD44-expressing macrophages are not as prominent in the colon of transfer colitis mice and the amount of hyaluronic acid in their colon tissue is not changing during the course of inflammation, suggests CD44^+^ macrophages and HA as the main structures for VSOP binding and accumulation. In DSS-induced colitis, VSOPs seem to be taken up by CD44 expressing macrophages in the periphery, which then get recruited to the inflamed colon by binding to the endothelial cells expressing high amounts of HA. This is supported by the clustering of VSOPs with CD31^+^ endothelial cells additional to CD44^+^ macrophages in colon tissue of DSS-treated mice. These results emphasize that VSOP accumulation seems to be dependent on specific ECM structures of the respective tissue with the HA content potentially playing a major role. As dysregulated production of HA is proved to be associated with IBD (Petrey and de la Motte, 2019) it represents an attractive target. Nevertheless, it cannot be excluded that VSOP accumulation is based on other interactions which are not displayed by the used models. Further studies including models of fibrosis like the chronic *Salmonella enterica* infection (Grassl et al., 2008) or chronic DSS colitis (Dieleman et al., 1994) would help to gain further insights on the underlying mechanisms and to cover the variety of pathophysiological features in IBD patients. For further application of VSOPs in a clinical setting, among other things, toxicity and biocompatibility of the contrast agent needs to be critically assessed. Currently, no clinical studies have been conducted so far. *In-vitro* labeling of human adipose tissue derived from stromal cells with VSOPs shows no influence on the physiological function of the cells. Preclinical *in-vivo* characterization in pigs and mice confirmed a good tolerance and safety profile (Wagner et al., 2002; Radeloff et al., 2020). Preliminary studies done by Wagner et al. measured a LD_50_ (lethal dose) in mice of >17.9 mmol Fe per kg of body weight (Wagner et al., 2002). In summary, the data indicated successful imaging of early inflammatory changes in DSS-induced colitis is feasible by VSOP-enhanced T_2_*w MRI. VSOP binding might depend on the HA content in respective tissues and VSOPs accumulate in CD44-expressing macrophages and endothelial cells of inflamed tissue. The study presented herein suggests VSOPs as a candidate for early diagnosis of inflammatory changes and an altered ECM profile in IBD.

## Data Availability

The raw data supporting the conclusion of this article will be made available by the authors, without undue reservation.
