# Supplementary material for: Visualization of Inflammation in Experimental Colitis by Magnetic Resonance Imaging Using Very Small Superparamagnetic Iron Oxide Particles
Source: Front Physiol. 2022 Jul 12;13:862212. doi: 10.3389/fphys.2022.862212 (PMC9315402; doi:10.3389/fphys.2022.862212)
Supplement: Supplementary file 1 [file Presentation1.PPTX]

## Slide 1
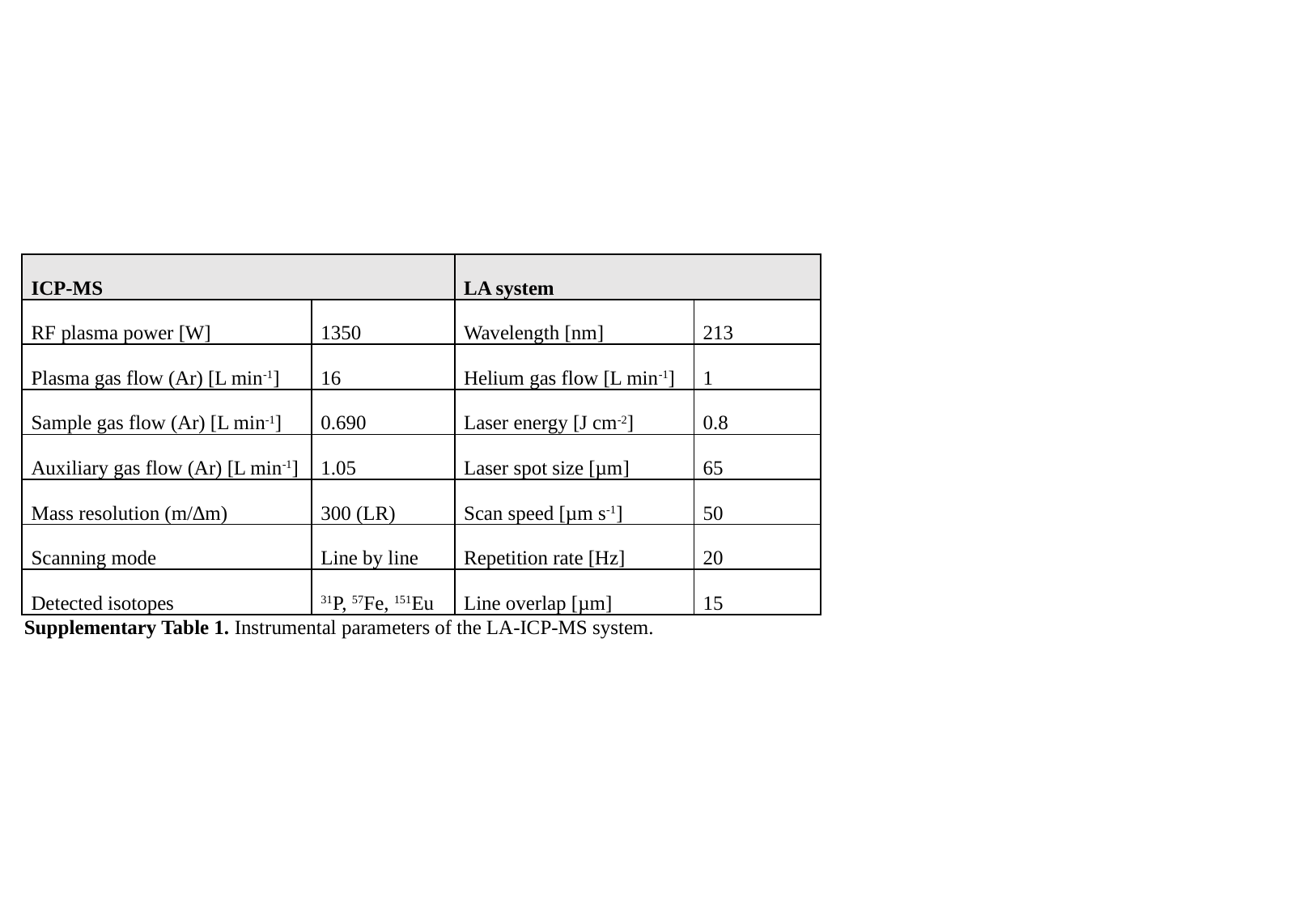

| ICP-MS | | LA system | |
| --- | --- | --- | --- |
| RF plasma power [W] | 1350 | Wavelength [nm] | 213 |
| Plasma gas flow (Ar) [L min-1] | 16 | Helium gas flow [L min-1] | 1 |
| Sample gas flow (Ar) [L min-1] | 0.690 | Laser energy [J cm-2] | 0.8 |
| Auxiliary gas flow (Ar) [L min-1] | 1.05 | Laser spot size [µm] | 65 |
| Mass resolution (m/∆m) | 300 (LR) | Scan speed [µm s-1] | 50 |
| Scanning mode | Line by line | Repetition rate [Hz] | 20 |
| Detected isotopes | 31P, 57Fe, 151Eu | Line overlap [µm] | 15 |
Supplementary Table 1. Instrumental parameters of the LA-ICP-MS system.

## Slide 2
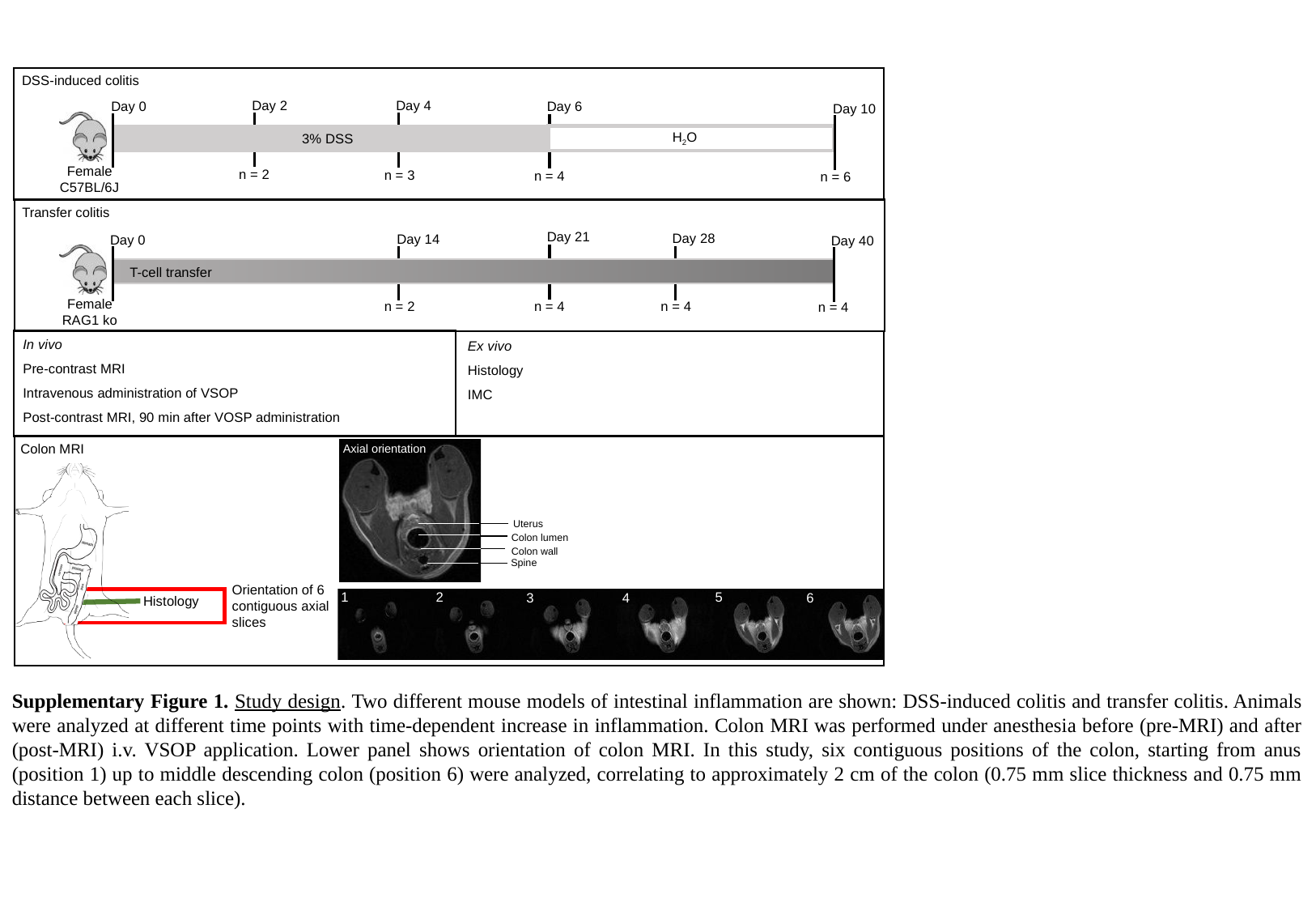

DSS-induced colitis
Day 2
Day 4
Day 6
Day 0
Day 10
DSS (3%)
n = 2
H2O
3% DSS
Female
C57BL/6J
n = 3
n = 4
n = 6
Transfer colitis
Day 21
Day 14
Day 0
Day 40
Day 28
T-cell transfer
Female
RAG1 ko
n = 4
n = 2
n = 4
n = 4
Colon MRI
Orientation of 6
contiguous axial slices
Histology
1
Day 28
In vivo
Pre-contrast MRI
Intravenous administration of VSOP
Post-contrast MRI, 90 min after VOSP administration
Ex vivo
Histology
IMC
Axial orientation
Uterus
Colon lumen
Colon wall
Spine
1
2
5
3
4
6
Supplementary Figure 1. Study design. Two different mouse models of intestinal inflammation are shown: DSS-induced colitis and transfer colitis. Animals were analyzed at different time points with time-dependent increase in inflammation. Colon MRI was performed under anesthesia before (pre-MRI) and after (post-MRI) i.v. VSOP application. Lower panel shows orientation of colon MRI. In this study, six contiguous positions of the colon, starting from anus (position 1) up to middle descending colon (position 6) were analyzed, correlating to approximately 2 cm of the colon (0.75 mm slice thickness and 0.75 mm distance between each slice).

## Slide 3
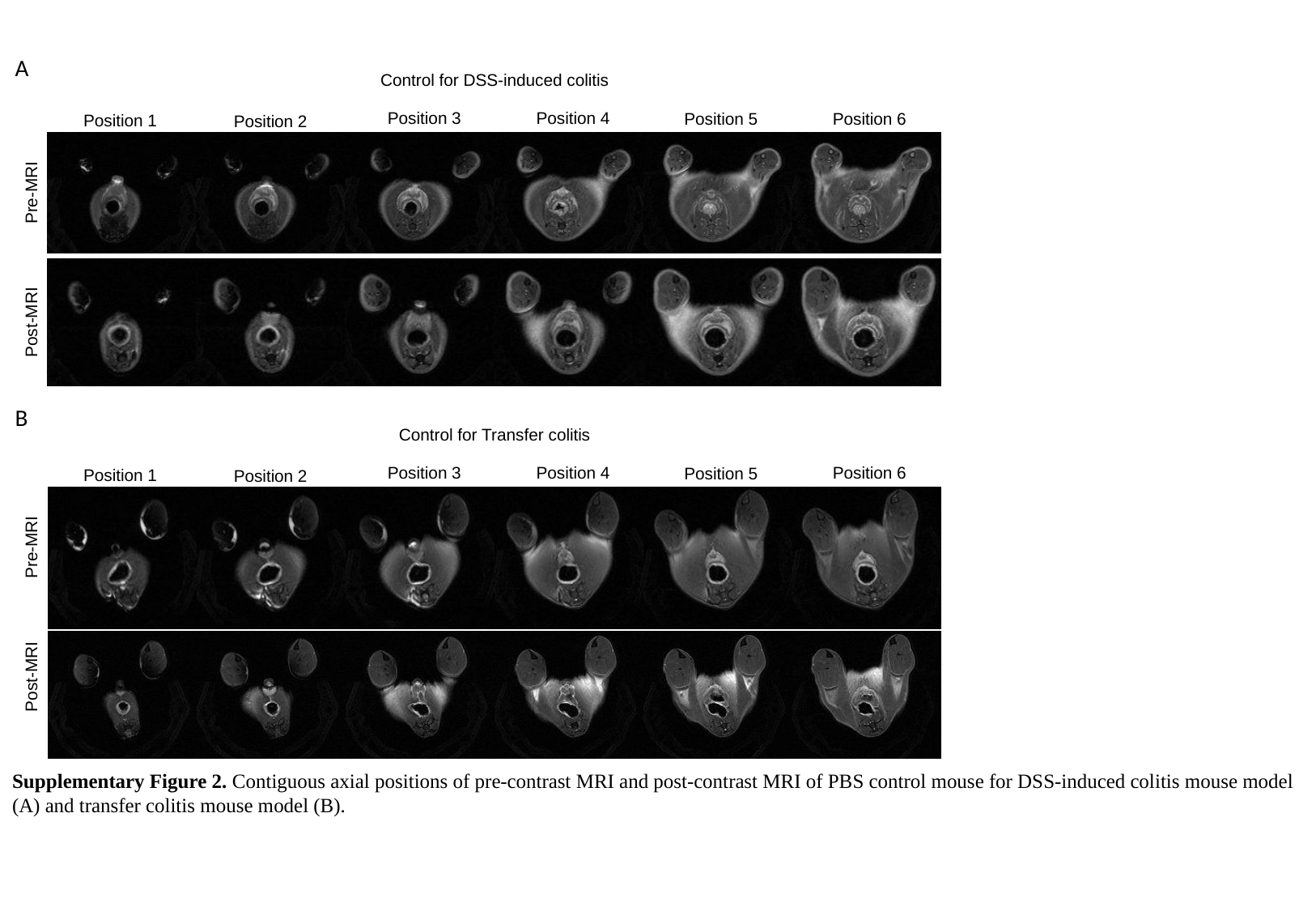

A
Control for DSS-induced colitis
Position 3
Position 4
Position 6
Position 5
Position 1
Position 2
Pre-MRI
Post-MRI
B
Control for Transfer colitis
Position 3
Position 4
Position 6
Position 5
Position 1
Position 2
Pre-MRI
Post-MRI
Supplementary Figure 2. Contiguous axial positions of pre-contrast MRI and post-contrast MRI of PBS control mouse for DSS-induced colitis mouse model (A) and transfer colitis mouse model (B).

## Slide 4
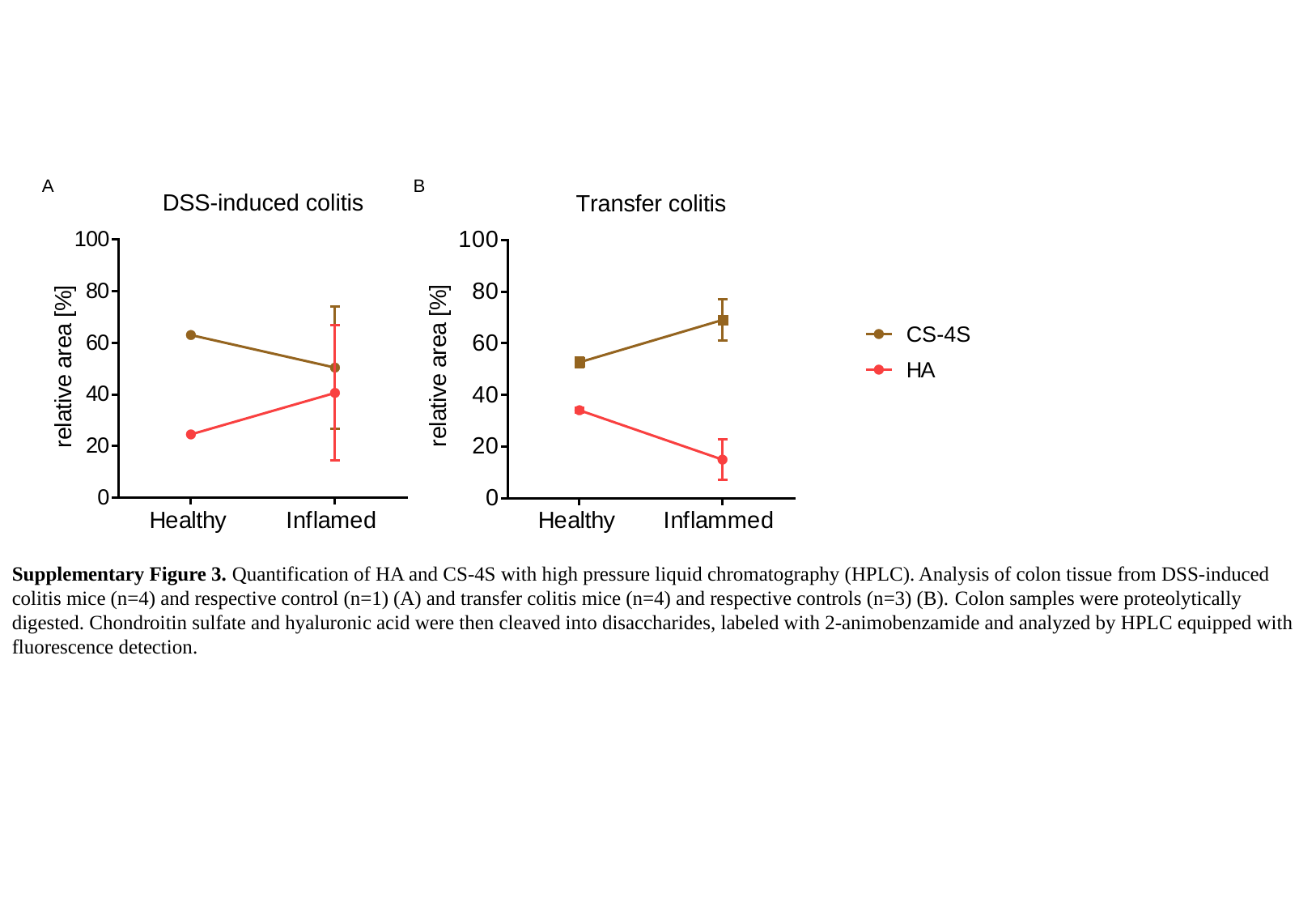

A
B
Supplementary Figure 3. Quantification of HA and CS-4S with high pressure liquid chromatography (HPLC). Analysis of colon tissue from DSS-induced colitis mice (n=4) and respective control (n=1) (A) and transfer colitis mice (n=4) and respective controls (n=3) (B). Colon samples were proteolytically digested. Chondroitin sulfate and hyaluronic acid were then cleaved into disaccharides, labeled with 2-animobenzamide and analyzed by HPLC equipped with fluorescence detection.

## Slide 5
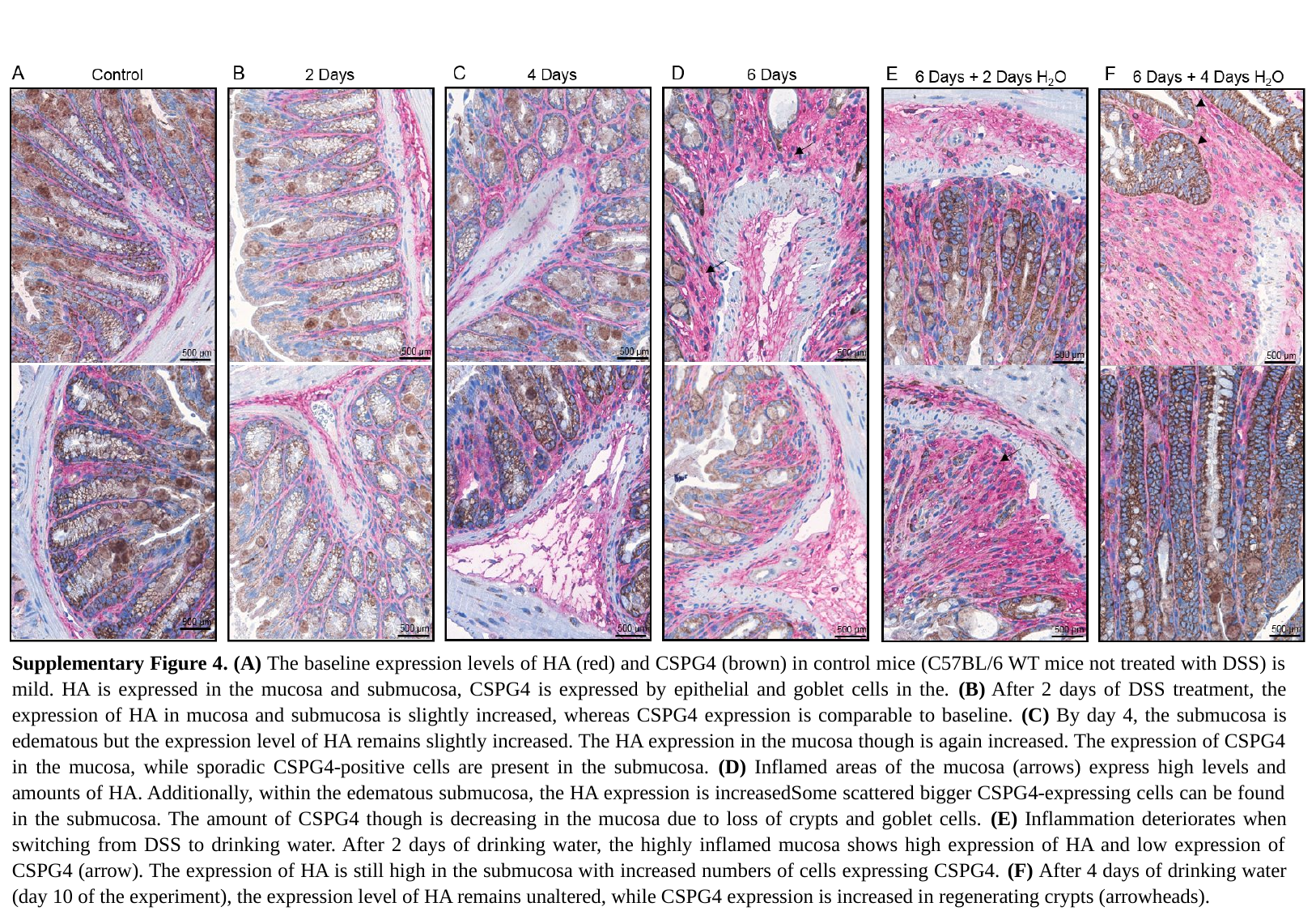

Supplementary Figure 4. (A) The baseline expression levels of HA (red) and CSPG4 (brown) in control mice (C57BL/6 WT mice not treated with DSS) is mild. HA is expressed in the mucosa and submucosa, CSPG4 is expressed by epithelial and goblet cells in the. (B) After 2 days of DSS treatment, the expression of HA in mucosa and submucosa is slightly increased, whereas CSPG4 expression is comparable to baseline. (C) By day 4, the submucosa is edematous but the expression level of HA remains slightly increased. The HA expression in the mucosa though is again increased. The expression of CSPG4 in the mucosa, while sporadic CSPG4-positive cells are present in the submucosa. (D) Inflamed areas of the mucosa (arrows) express high levels and amounts of HA. Additionally, within the edematous submucosa, the HA expression is increasedSome scattered bigger CSPG4-expressing cells can be found in the submucosa. The amount of CSPG4 though is decreasing in the mucosa due to loss of crypts and goblet cells. (E) Inflammation deteriorates when switching from DSS to drinking water. After 2 days of drinking water, the highly inflamed mucosa shows high expression of HA and low expression of CSPG4 (arrow). The expression of HA is still high in the submucosa with increased numbers of cells expressing CSPG4. (F) After 4 days of drinking water (day 10 of the experiment), the expression level of HA remains unaltered, while CSPG4 expression is increased in regenerating crypts (arrowheads).

## Slide 6
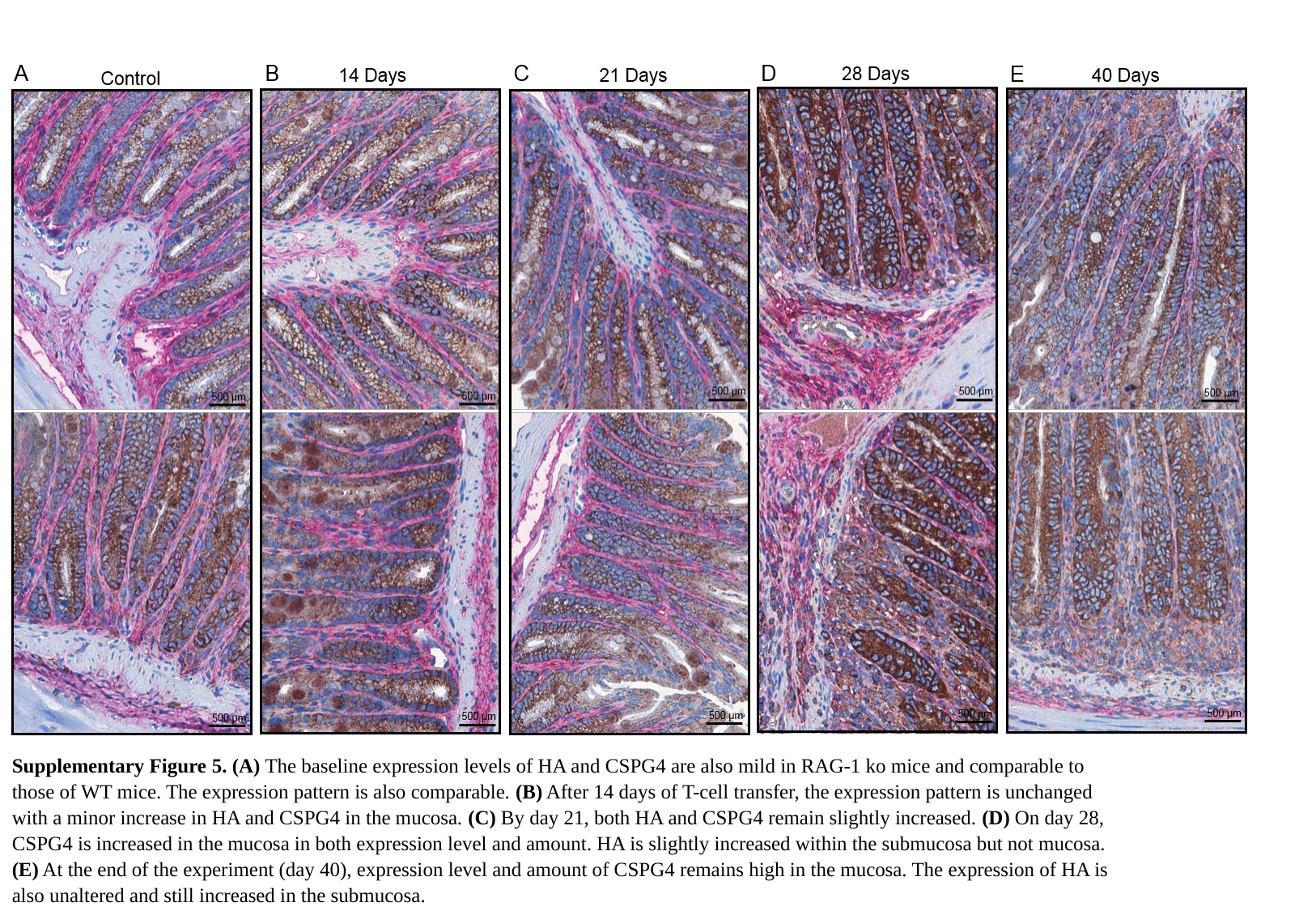

Supplementary Figure 5. (A) The baseline expression levels of HA and CSPG4 are also mild in RAG-1 ko mice and comparable to those of WT mice. The expression pattern is also comparable. (B) After 14 days of T-cell transfer, the expression pattern is unchanged with a minor increase in HA and CSPG4 in the mucosa. (C) By day 21, both HA and CSPG4 remain slightly increased. (D) On day 28, CSPG4 is increased in the mucosa in both expression level and amount. HA is slightly increased within the submucosa but not mucosa. (E) At the end of the experiment (day 40), expression level and amount of CSPG4 remains high in the mucosa. The expression of HA is also unaltered and still increased in the submucosa.

## Slide 7
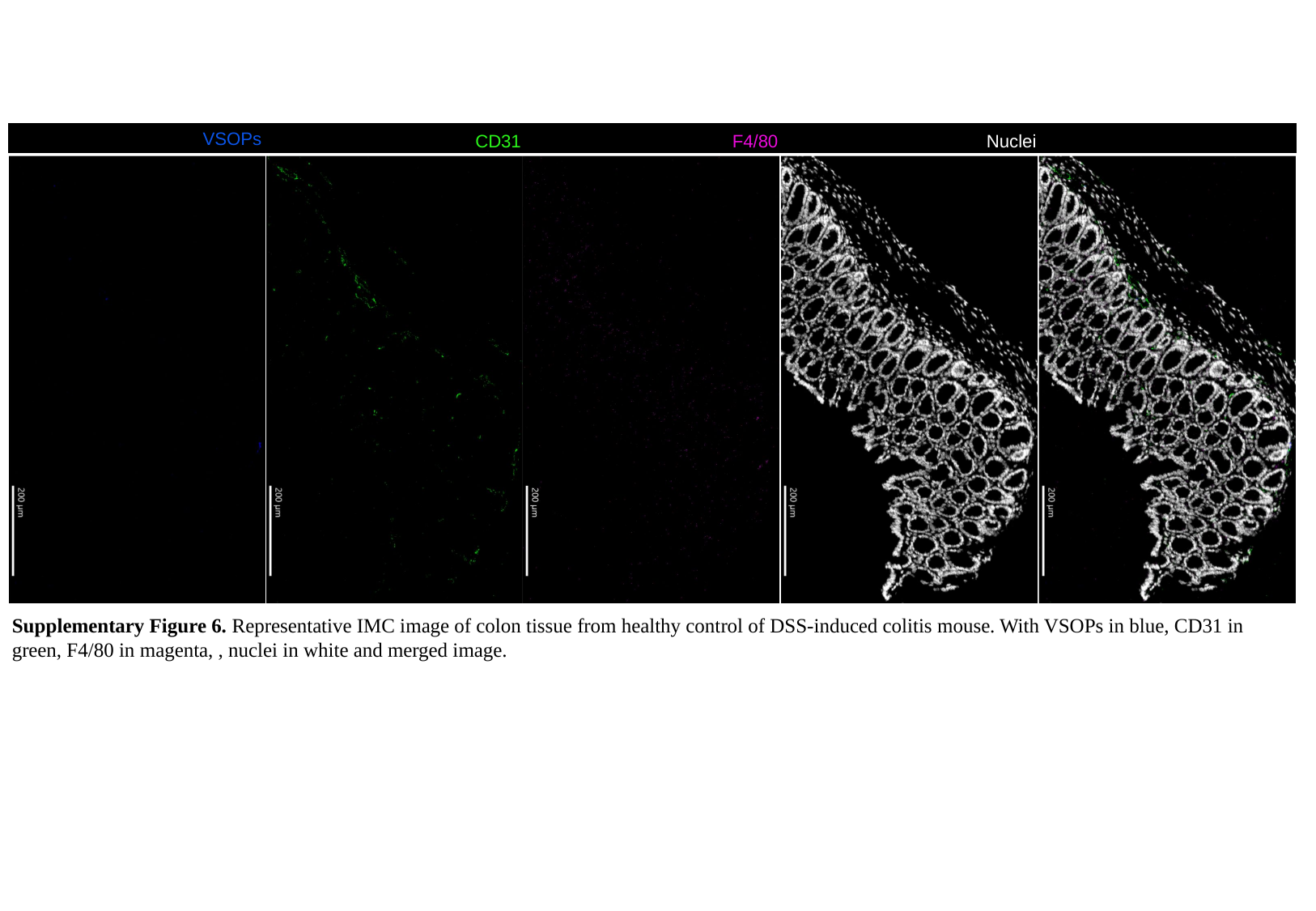

VSOPs
CD31
F4/80
Nuclei
Supplementary Figure 6. Representative IMC image of colon tissue from healthy control of DSS-induced colitis mouse. With VSOPs in blue, CD31 in green, F4/80 in magenta, , nuclei in white and merged image.

## Slide 8
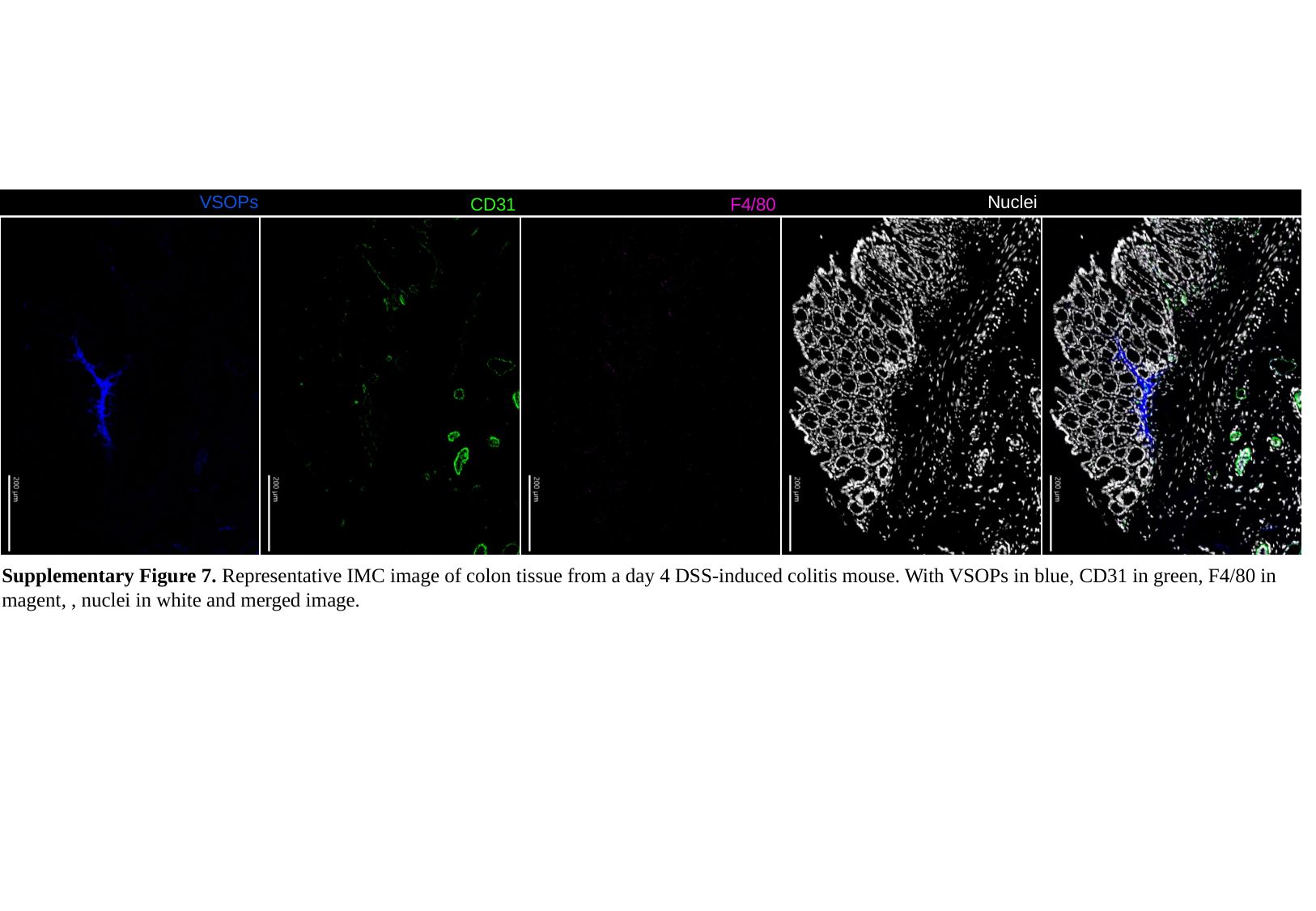

VSOPs
Nuclei
CD31
F4/80
Supplementary Figure 7. Representative IMC image of colon tissue from a day 4 DSS-induced colitis mouse. With VSOPs in blue, CD31 in green, F4/80 in magent, , nuclei in white and merged image.

## Slide 9
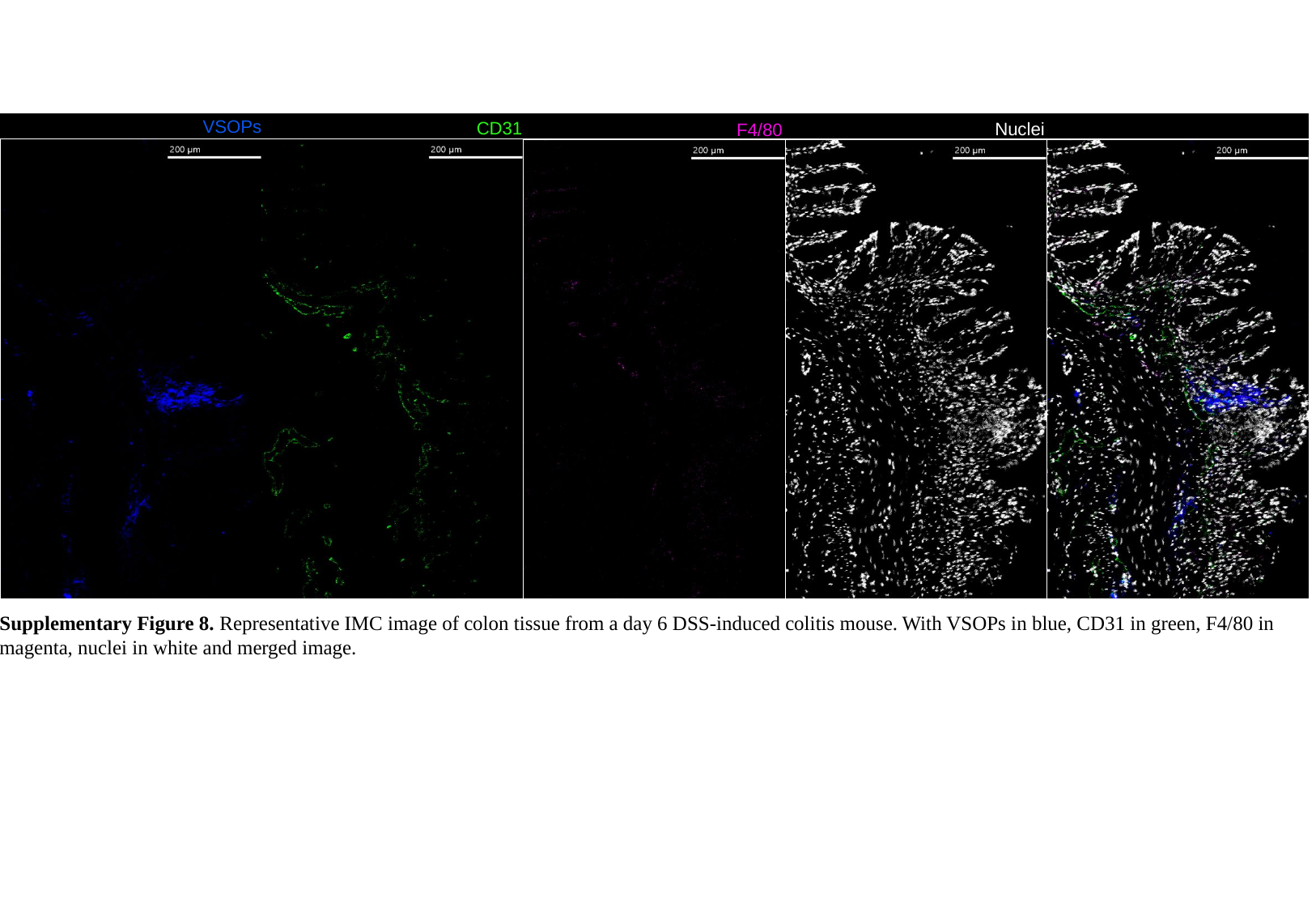

VSOPs
CD31
Nuclei
F4/80
Supplementary Figure 8. Representative IMC image of colon tissue from a day 6 DSS-induced colitis mouse. With VSOPs in blue, CD31 in green, F4/80 in magenta, nuclei in white and merged image.

## Slide 10
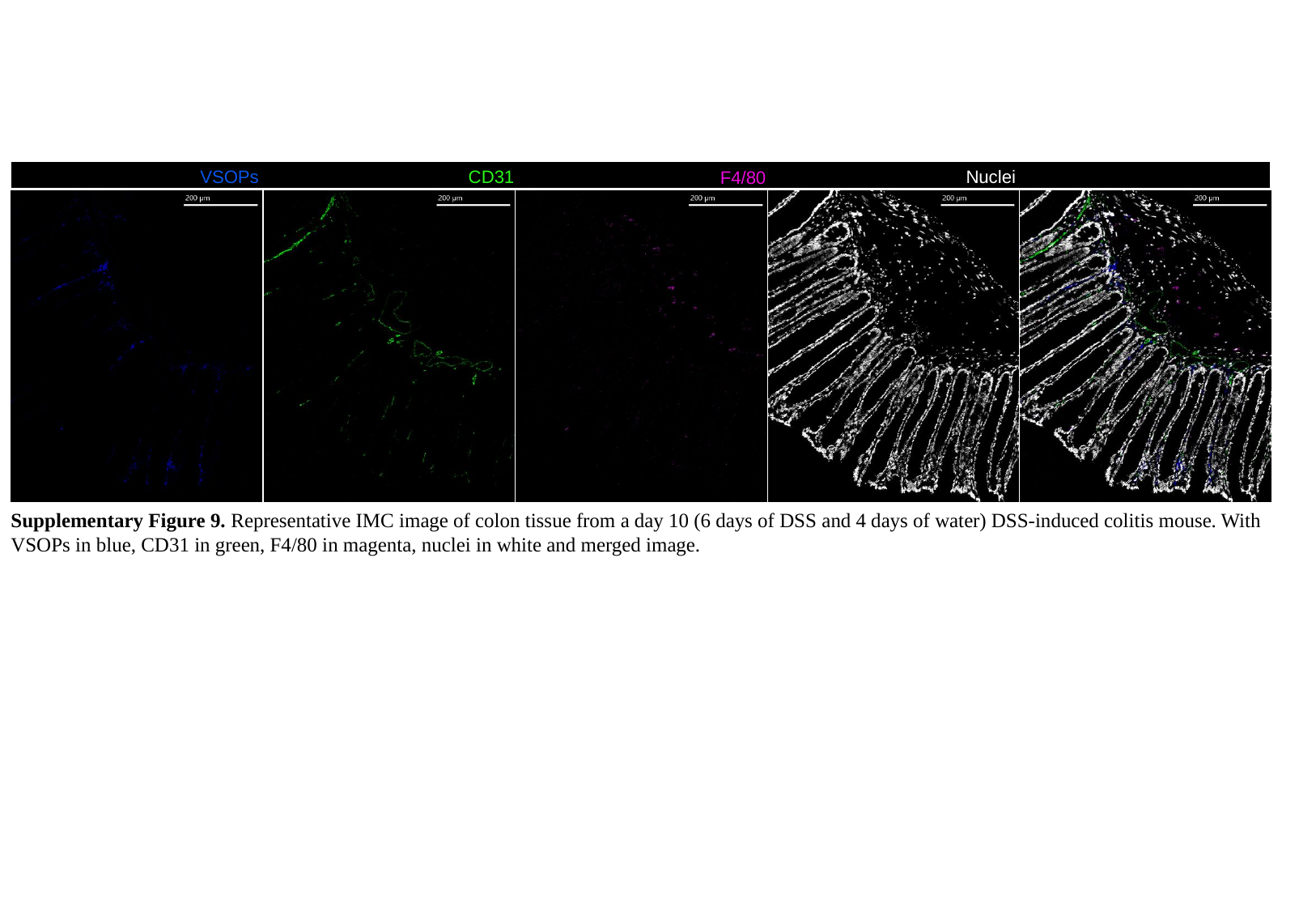

VSOPs
CD31
Nuclei
F4/80
Supplementary Figure 9. Representative IMC image of colon tissue from a day 10 (6 days of DSS and 4 days of water) DSS-induced colitis mouse. With VSOPs in blue, CD31 in green, F4/80 in magenta, nuclei in white and merged image.

## Slide 11
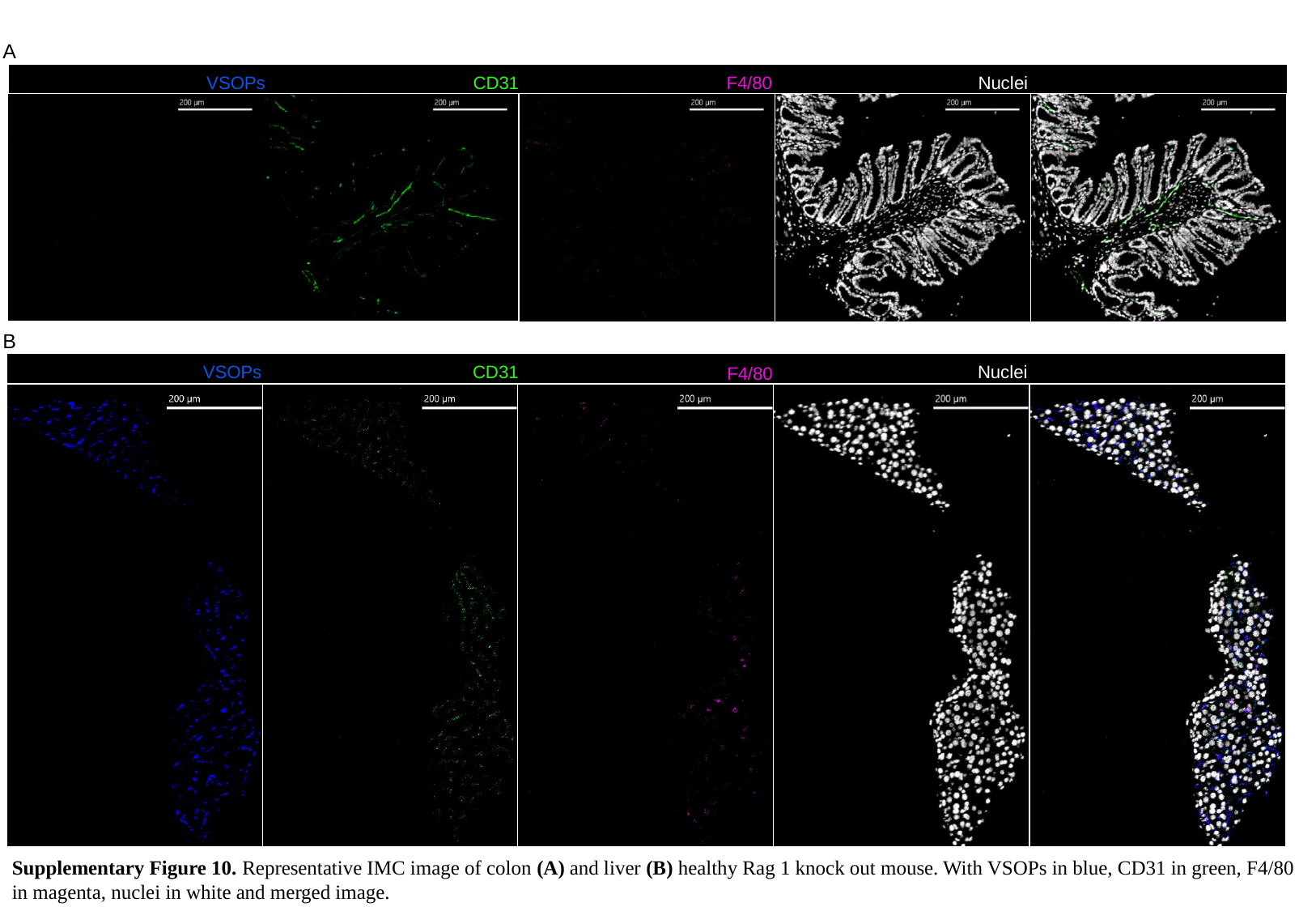

A
VSOPs
CD31
F4/80
Nuclei
B
VSOPs
CD31
Nuclei
F4/80
Supplementary Figure 10. Representative IMC image of colon (A) and liver (B) healthy Rag 1 knock out mouse. With VSOPs in blue, CD31 in green, F4/80 in magenta, nuclei in white and merged image.

## Slide 12
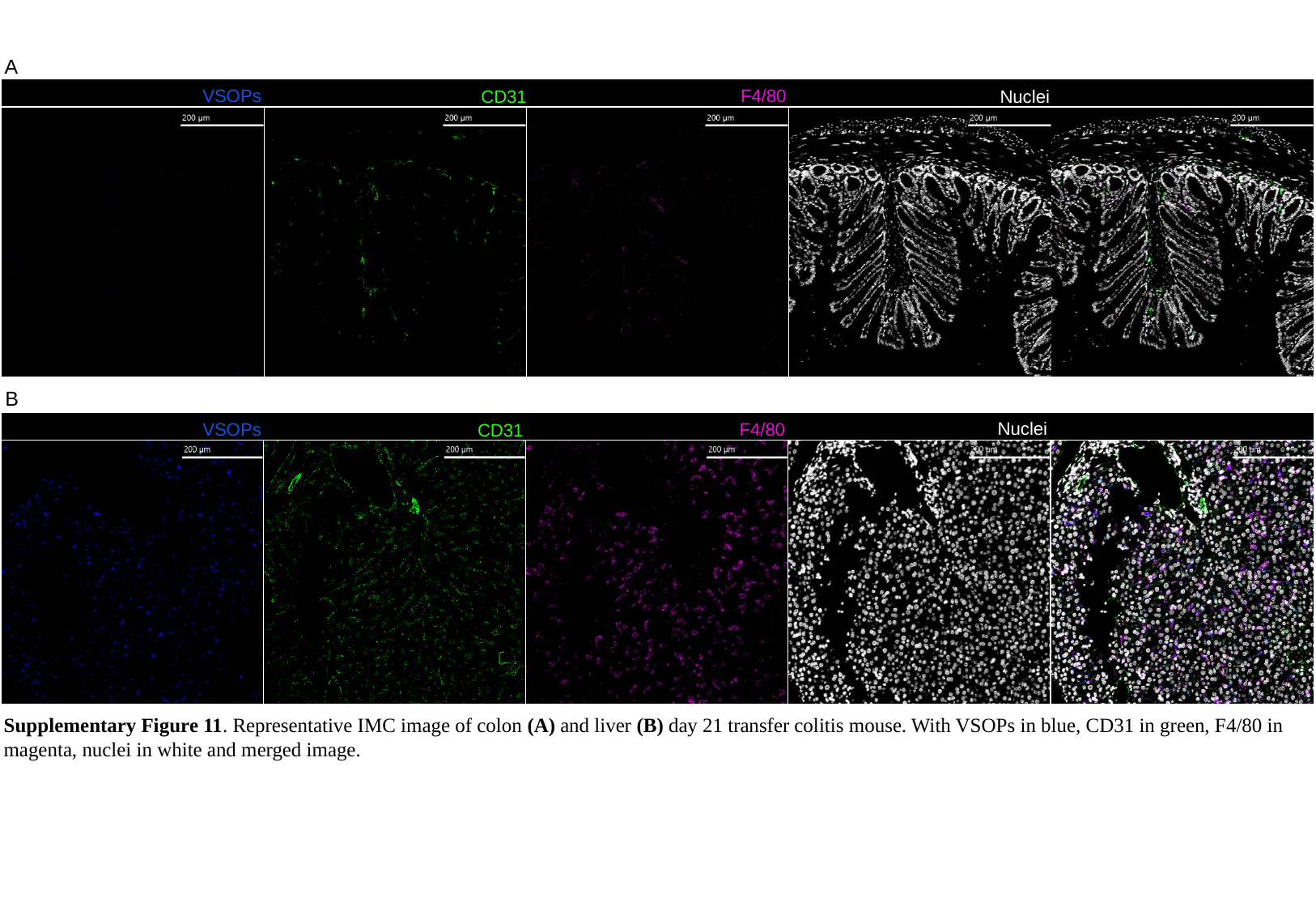

A
VSOPs
F4/80
Nuclei
CD31
B
Nuclei
VSOPs
F4/80
CD31
Supplementary Figure 11. Representative IMC image of colon (A) and liver (B) day 21 transfer colitis mouse. With VSOPs in blue, CD31 in green, F4/80 in magenta, nuclei in white and merged image.

## Slide 13
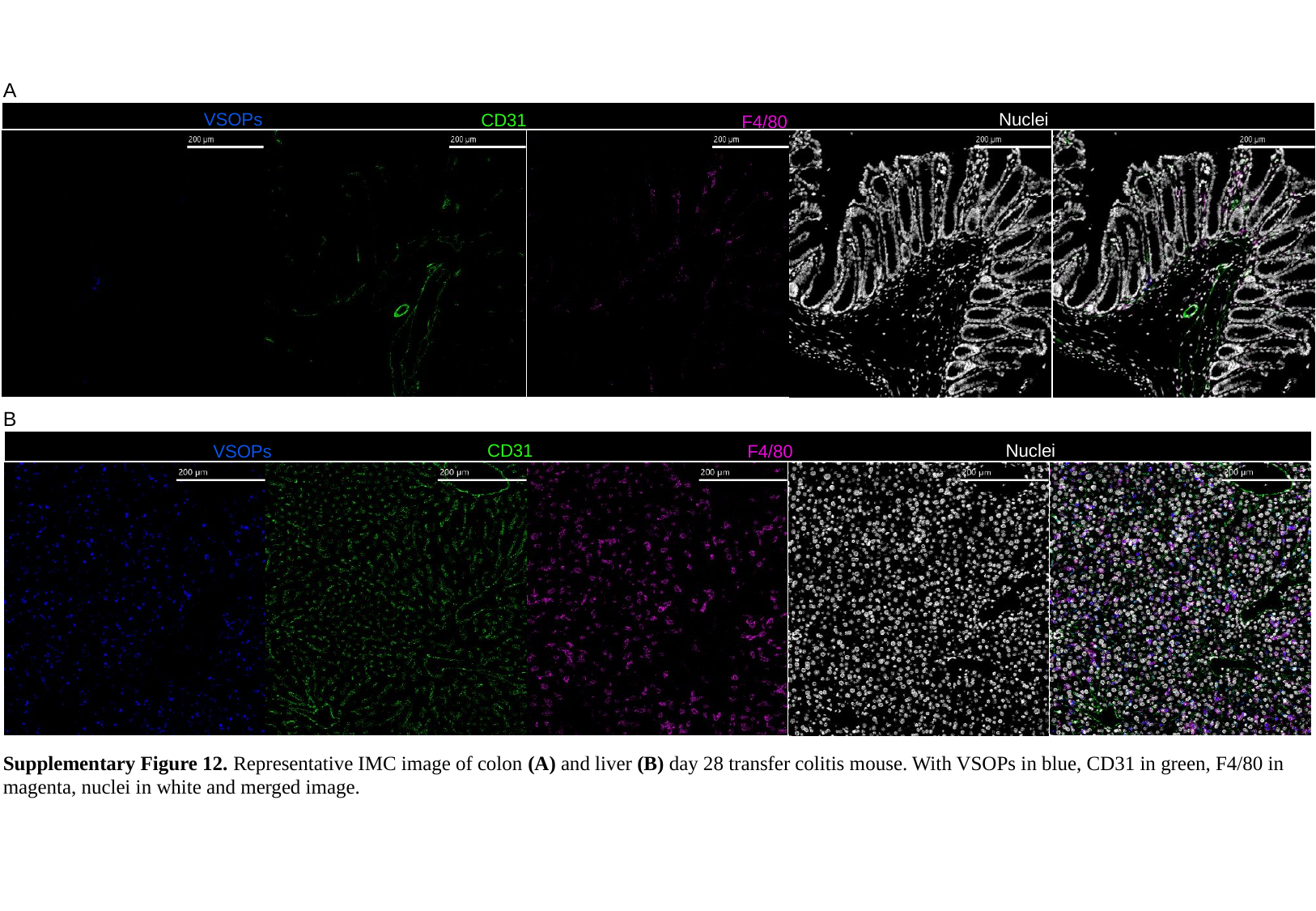

A
VSOPs
Nuclei
CD31
F4/80
B
CD31
Nuclei
VSOPs
F4/80
Supplementary Figure 12. Representative IMC image of colon (A) and liver (B) day 28 transfer colitis mouse. With VSOPs in blue, CD31 in green, F4/80 in magenta, nuclei in white and merged image.

## Slide 14
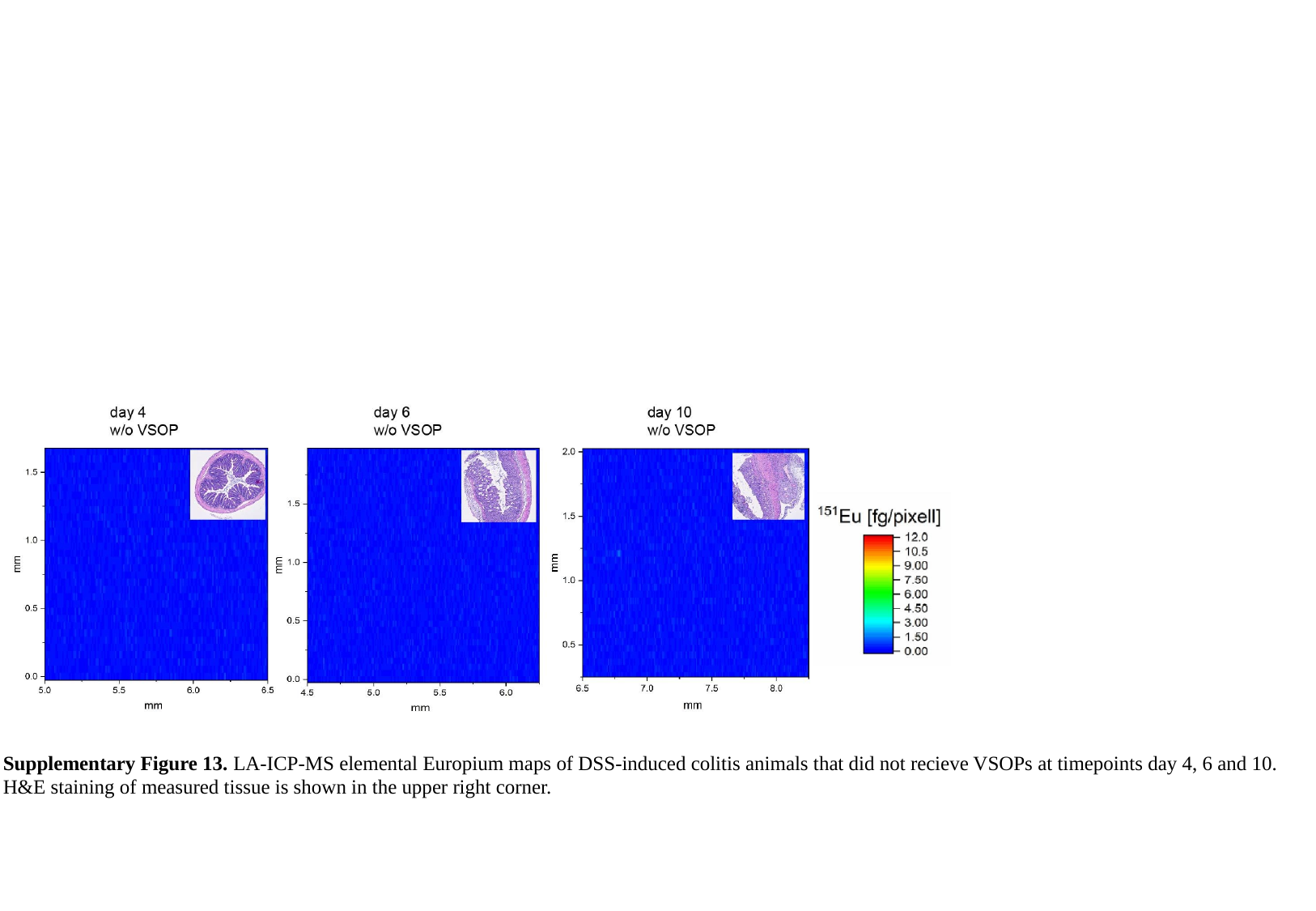

Supplementary Figure 13. LA-ICP-MS elemental Europium maps of DSS-induced colitis animals that did not recieve VSOPs at timepoints day 4, 6 and 10. H&E staining of measured tissue is shown in the upper right corner.
